# Multifractal Desynchronization of the Cardiac Excitable Cell Network During Atrial Fibrillation. II. Modeling

**DOI:** 10.3389/fphys.2019.00480

**Published:** 2019-04-24

**Authors:** Guillaume Attuel, Evgeniya Gerasimova-Chechkina, Françoise Argoul, Hussein Yahia, Alain Arneodo

**Affiliations:** ^1^Geometry and Statistics in Acquisition Data, Centre de Recherche INRIA, Talence, France; ^2^Laboratory of Physical Foundation of Strength, Institute of Continuous Media Mechanics UB RAS, Perm, Russia; ^3^Laboratoire Ondes et Matières d'Aquitaine, Université de Bordeaux, UMR 5798, CNRS, Talence, France

**Keywords:** atrial fibrillation, modeling, excitable cell network, kinetics of gap junction channel, multifractal analysis, intermittent dynamics

## Abstract

In a companion paper (I. Multifractal analysis of clinical data), we used a wavelet-based multiscale analysis to reveal and quantify the multifractal intermittent nature of the cardiac impulse energy in the low frequency range ≲ 2Hz during atrial fibrillation (AF). It demarcated two distinct areas within the coronary sinus (CS) with regionally stable multifractal spectra likely corresponding to different anatomical substrates. The electrical activity also showed no sign of the kind of temporal correlations typical of cascading processes across scales, thereby indicating that the multifractal scaling is carried by variations in the large amplitude oscillations of the recorded bipolar electric potential. In the present study, to account for these observations, we explore the role of the kinetics of gap junction channels (GJCs), in dynamically creating a new kind of imbalance between depolarizing and repolarizing currents. We propose a one-dimensional (1D) spatial model of a denervated myocardium, where the coupling of cardiac cells fails to synchronize the network of cardiac cells because of abnormal transjunctional capacitive charging of GJCs. We show that this non-ohmic nonlinear conduction 1D modeling accounts quantitatively well for the “multifractal random noise” dynamics of the electrical activity experimentally recorded in the left atrial posterior wall area. We further demonstrate that the multifractal properties of the numerical impulse energy are robust to changes in the model parameters.

## 1. Introduction

Atrial fibrillation (AF) is the most common sustained tachyarrhythmia encountered in clinical practice (Nattel and Harada, 2014). It is sometimes not diagnosed until the occurrence of a severe complication such as embolic stroke. Often associated with heart disease, clinical investigations do not always uncover any preexisting cardiovascular comorbidity (idiopathic or lone AF). Physiologically, current understanding of the onset and perpetuation of most tachyarrhythmias including AF presumes the involvement of circuit reentries. This scenario was established historically from the observation of reciprocating rhythms initiated in the atrio-ventricular node via the fast and slow pathways of impulse conduction. Atrial flutter and regular tachycardias were thus inferred to be rooted in circling conduction pathways, as going around anatomical obstacles or scars for example. Tachyarrhythmias may also spontaneously evolve toward more irregular arrhythmias such as AF. Experiments show that AF can be induced by *in situ* injection of toxins like aconitine as well as by ectopic stimulation, i.e., under extreme conditions enforcing local functional changes of the excitable conducting substrate. AF may then persist independently of the inciting protocol (Macfarlane et al., 2011; Zipes et al., 2017). These observations paved the way to the concept of multiple circuit reentries, not necessarily linked to the anatomy but to a vulnerable atrial substrate because of functional dispersion in space and time (such as non uniform dispersion of refractoriness) (Moe and Abildskov, 1959; Moe et al., 1964; Allessie et al., 1977; Attuel et al., 1989; Winfree, 1989). But clinically, the question remained whether abnormal conducting pathways and ectopic triggers do stabilize AF. In that respect, intervention procedures were developed either to surgically create an electrical maze in the atria or, in a less invasive and safer way, to isolate abnormal ectopic activity as found in the pulmonary veins areas by radio frequency ablation. Both procedures led to high clinical success rates in stopping paroxysmal AF (Cox et al., 1991; Ha¨ıssaguerre et al., 1998). Unfortunately, the diverse procedures instigated since then remain suboptimal because the risk of relapse increases with time, and the disease often evolves toward a chronic state (Ganesan et al., 2013; Takigawa et al., 2014).

Cardiomyocytes belong to the family of excitable cells which are ubiquitous in animals and plants (Hille, 2001). They are distinguishable from non-excitable cells by their ability to reach an electrically depolarized state of their extra-cellular phospolipid bi-layer membranes. Action potentials (APs) correspond to cycle events in which the membrane reaches a depolarized state before relaxing back to the polarized rest state. In the wake of the seminal work by Hodgkin and Huxley on the giant squid axon AP (Hodgkin and Huxley, 1952a,b), a cardiac impulse is similarly described by the nonlinear coupling between a diffusing activator, the electric potential across the excitable cell membrane, and a non-diffusing inhibitor, the overall ion currents permeating through the membrane (Noble, 1962, 1965; Beeler and Reuter, 1977; Plonsey and Barr, 2007; Fenton and Cherry, 2008; Macfarlane et al., 2011). This nonlinearity underlies the fact that the AP amplitudes depend very little on the intensity of the exciting perturbation, provided they are suprathreshold. Various transmembrane proteins selectively allow some solutes to permeate. Leaking (potassium) channels are balanced by (sodium-potassium) pump exchangers forcing the cell membrane into a negatively polarized state, which compensates for the hypertonic activity of internally sequestered vital substances (Tosteson and Hoffman, 1960; Armstrong, 2003). Excitable cells take advantage of this situation to generate electrical signals. Their plasma membrane incorporates a large number of ion channels, sensitive to various other species such as e.g., calcium. They are proteins forming pores that greatly facilitate ion transport down electrochemical gradients. Ion channels act as voltage dependent gates and their reaction rates reflect the height of the free energy barrier separating the open and closed conformation states (Hille, 2001). The membrane depolarizes in a few milliseconds to a near Nernst-Planck resting equilibrium, as for instance triggered by a supra-threshold electrical stimulus. In the heart, in addition, each cardiomyocyte cycle lasts a definite amount of time, typically a few hundreds milliseconds, incorporating a refractory period during which re-excitation is impossible.

Electric pulses travel by ohmic conduction within a single cell membrane, whereas the transport of these pulses across adjacent cardiac cells is ensured by gap junction channels (GJCs). These GJCs connect adjacent cardiac cells along a preferred longitudinal direction, each via tight assemblies of hundreds to thousands of pores, themselves formed by two hemichannels of six bound proteins called connexins. Also found in nearly every connected animal tissues, and on the contrary to ion channels, GJCs are wide and open at rest, thereby prominently contributing to homeostasis, chemical messaging, and electrical permeability throughout the whole network of connected cells (Weidmann, 1966; Severs, 1990; Kumar and Gilula, 1996; Harris, 2001; Evans and Martin, 2002). The reduction of connexin genetic expression was shown to be source of conduction inhomogeneities increasing susceptibility to arrhythmias in ventricles and atria, in various animal models and in humans with diverse heart conditions, potentially causing sudden cardiac death (van der Velden et al., 2000; Dupont et al., 2001; van der Velden and Jongsma, 2002; Ausma et al., 2003; Danik et al., 2004; Severs et al., 2008). Recent studies also suggest that missense mutations in connexin encoding genes predispose to AF (Gollob et al., 2006), whereas connexin gene transfer plays a protective role in preventing sustained AF (Bikou et al., 2011; Igarashi et al., 2012).

The phenomenology of irregular arrhythmias is classically interpreted as emanating from the chaotic dynamics of excitable reaction-diffusion systems in which ohmic conduction is assumed. Typical routes to deterministic chaos with specific rhythms were theoretically identified in simple models based on cell cycle phase resetting (Glass and Mackey, 1979, 1987; Guevara et al., 1981; Guevara and Glass, 1982). In particular, period doubling bifurcations in the dynamics of excitable pulses propagating on a ring (Courtemanche et al., 1993) were put forward as a mechanism prior to the onset of AF. More complete ion channel models such as the historical Beeler-Reuter model (Beeler and Reuter, 1977), were shown to succeed in generating generic spatio-temporal patterns (Jensen et al., 1984; Chialvo et al., 1990; Fenton and Cherry, 2008). Spiral waves in 2D spatial dimensions (theoretically scroll waves in 3D) were observed experimentally during ventricular tachycardia as well as before the onset of fibrillation (Pertsov et al., 1993; Gray et al., 1995; Garfinkel et al., 1997; Zipes et al., 2017). These spiral waves can be seen as focal wave trains swirling periodically around the analog of the “leading circle” at their core (Allessie et al., 1977; Winfree, 1989). More recently, cellular automata on a two-dimensional square lattice was used to demonstrate that phase-dependent spiral attenuation could reproduce wave propagation in excitable media of myocardial cells (de la Casa et al., 2007a,b). Within this framework, fibrillation was interpreted as the break-up of unstable spiral waves spoiling rhythmic regularity (Gerhardt et al., 1990; Ito and Glass, 1991; Bär and Eiswirth, 1993; Karma, 1993; Panfilov and Hogeweg, 1993; Bär and Brusch, 2004; Zipes et al., 2017). Other models based on nonlinear stochastic feedback mechanisms were also proposed to explain the regulation of cardiac dynamics (Ivanov et al., 1998), suggesting that it could be intrinsically random (physiological noise). To our knowledge, it has never been reported in cardiac models the existence of a multifractal intermittent dynamics of the cardiac impulse energy as observed experimentally over large time scales in our companion paper I (Attuel et al., 2017). In this study, we elaborate on a tentative interpretation of the observed intermittent dynamics during AF as the signature of synaptic plasticity. Typical individual GJC transition times between open and closed states were shown to be much longer than those of membrane polarization but compare well with membrane recovery time (≳ 100ms) (Spray et al., 1984; Neyton and Trautmann, 1986; Wang et al., 1992; Bukauskas and Verselis, 2004; Desplantez et al., 2007). Moreover, slow gating modulations have been evidenced due to cytoplasmic protons (low pH) and free calcium (Spray et al., 1984; Burt and Sray, 1988; Kumar and Gilula, 1996; Harris, 2001; Bukauskas and Verselis, 2004; Perrachia, 2004; Swietach et al., 2013). Thus, a perturbation of the GJC opening and closing due to electric charge may induce some time lag or advance in the activation of the cell, slowing down or boosting the propagation of the AP, even impeding or reversing it (after reexcitation), resulting in a local departure from ohmic conduction law.

Here, we propose a mathematical model of cardiac cell excitability which includes their dynamical coupling by GJC kinetics. As (i) gap junctions electrically bind cardiac cells preferentially along their elongated direction (Severs, 1990; Evans and Martin, 2002)), and (ii) in the left atrial lateral wall area, the CS has a thin surrounding muscular structure traversed by myocardial strands (Ho et al., 2012), we simply consider a one-dimensional (1D) spatial model to describe the transport of AP along and across myocardial cells via the temporal interplay of voltage-gated channels and GJCs. We show that (if probably not minimal) this 1D model robustly accounts for the intermittent modulation of cardiac pulse trains experimentally observed in the clinical data recorded in the left atrial posterior wall area of the CS (The study reported in Companion paper I (Attuel et al., 2017) was carried out with the recommendations of the International Cardiac Electrophysiological Service of public hospital CHU Haut-Lévêque, Pessac, France. The protocol for clinic research was approved by the Institutional Clinical Research and Ethics Committee: CPP (Comité de Protection des Personnes) and AFSSaPS (Agence Française de Sécurité Sanitaire des Produits de Santé). All patients involved gave written informed consent to the investigation of data. For this specific investigation of the data, the authors accessed fully anonymized and de-identified data.

## 2. Model and Numerical Data

To reproduce the spatio-temporal multifractal intermittent dynamics of voltage signals collected from the CS of patients with chronic AF (companion paper I Attuel et al., 2017), we propose the following system of four nonlinearly coupled partial differential equations (PDEs) (Attuel et al., 2016):

(1) $$\begin{array}{l} \{\begin{array}{c} c_{m}\frac{\partial }{\partial t}U_{m}=\text{F}\left(U_{m},w_{m}\right)-c_{g}^{-1}\frac{\partial }{\partial x}\left(g\rho_{g}\right)+\kappa \frac{\partial^{2}}{\partial x^{2}}U_{m},\\ \frac{\partial }{\partial t}w_{m}=\text{G}\left(U_{m},w_{m}\right),\\ \frac{\partial }{\partial t}g=\omega^{2}\rho_{g}-\nu_{1}g,\\ \frac{\partial }{\partial t}\rho_{g}=-g\frac{\partial }{\partial x}U_{m}-\nu_{2}\rho_{g}, \end{array} \end{array}$$

where *U*_*m*_(*x, t*) is the membrane electric potential drop, *w*_*m*_(*x, t*) the ionic channel gating variable, *g*(*x, t*) the gap junction conductance deviation from normal, and ρ_*g*_(*x, t*) the gap junction capacitive charge density. *w*_*m*_(*x, t*) is generally a vector of state variables describing the generation of the APs that is related to the intracellular concentration variations of different ions (Na^+^, K^+^, Ca^2+^, Cl^−^). As explained in section 2.1, the kinetics of the voltage gated channels will be described by the simplified FitzHugh-Nagumo model (FitzHugh, 1962; Nagumo et al., 1962; Izhikevich and FitzHugh, 2006). Let us note that, as compared to previous modeling attempts, our model (Equation 1) lies on the assumption that the gap junction conductance is not static, and that the GJC gating is driven by both the local transmembrane field produced by the charging of the gap junction and its conductance (nonlinear coupling term). The first two equations correspond to the standard mono-domain model for cardiac AP conduction, in which an additional term for the GJC current has been introduced, responsible for an imbalance between depolarizing and repolarizing membrane currents. The last two equations describe the GJC conduction and capacitive charging.

### 2.1. Standard Modeling of Cardiac AP Conduction

**Figure 1:**
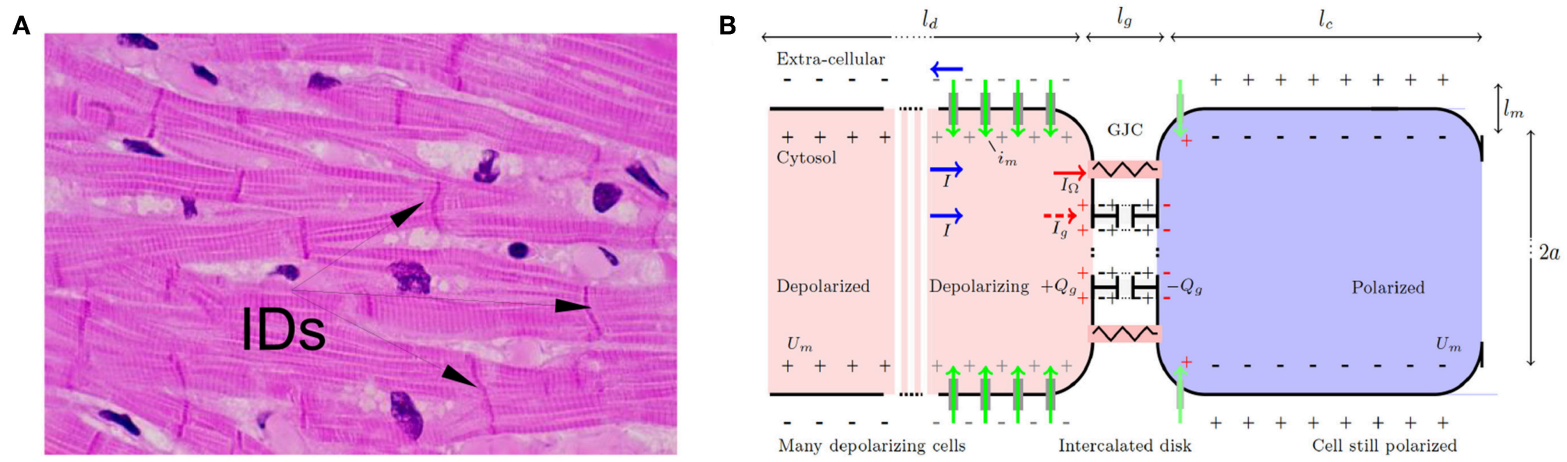
**(A)** Microscopic view of cardiac muscle cells (longitudinal section) stained with hematoxylin (nuclei) and eosin (cytoplasm). The myocardial cells can be recognized by their elongated aspect (50–100 μm long, with sections ~ 10μm), a longitudinal striated organization, multiple branchings and connections at their extremities via intercalated disks (IDs). **(B)** Schematic GJCs at the onset of depolarization. Depolarizing (resp. polarized) cells are stained in red (resp. blue). Surface charges are depicted with ± symbols, on the inner and outer side of the depolarizing (gray) and polarized (black) membranes and on the dysfunctional GJCs (red) with capacitive charge loading *Q*_*g*_ such that 〈*Q*_*g*_ 〉 = ∫ ρ_*g*_ d*x*. Voltage-gated channel ionic flows are marked with green vertical arrows. Normal GJCs: red resistor symbol, dysfunctional GJCs: gray capacitor symbol. Total currents *I* (blue horizontal arrow) circulate in opposite directions inside and outside the cell, splitting into the normally flowing ohmic current *I*_Ω_ (red), and residual closed GJCs current *I*_*g*_ (dashed red) building up charge. Typical membrane and intercalated disk dimensions are *l*_*m*_ ≈ *l*_*g*_ ∽ 5 nm.

Let us consider an idealized elongated fiber of excitable cells (Figure 1), along which a traveling depolarization front (AP upstroke) is classically modeled by a 1D cable equation, assuming Kirchhoff's law of conservation of currents (Plonsey and Barr, 2007; Niebur, 2008; Macfarlane et al., 2011):

(2) $$\begin{array}{l} \{\begin{array}{c} c_{m}\frac{\partial }{\partial t}U_{m}=\text{F}\left(U_{m},w_{m}\right)+\kappa \frac{\partial^{2}}{\partial x^{2}}U_{m},\\ \frac{\partial }{\partial t}w_{m}=\text{G}\left(U_{m},w_{m}\right), \end{array} \end{array}$$

where *U*_*m*_ (in V) is the electric potential across the cell membrane, *c*_*m*_ (in F/m) is the fiber's insulating membrane capacitance per unit length, κ = σ*S* (in Ω^−1^ × m) is an inverse resistance per unit length with σ the mono-domain conductivity^1^, and *S* (in m^2^) is the fiber cross-section. The coupling of the membrane electric potential *U*_*m*_ with inhibiting membrane currents responsible for repolarization by ion specific voltage-gated channels (Na^+^, K^+^, Ca^2+^, Cl^−^) is represented by the variable *w*_*m*_ (in A/m) in the nonlinear function **F**. The function **G** symbolizes the kinetics of the repolarizing voltage gated ion channels. One example of a boundary condition consists in imposing an external current stimulus *I*_*ext*_ (in A), possibly time dependent, at the *x* = 0 extremity of the cell array:

(3) $$\begin{array}{l} \kappa \frac{\partial }{\partial x}U_{m}\left(x=0\right)=-I_{ext}. \end{array}$$

A popular model for the nonlinear function **F**(*U*_*m*_, *w*_*m*_) in Equation 2 is the so-called FitzHugh-Nagumo (FHN) model (FitzHugh, 1962; Nagumo et al., 1962; Izhikevich and FitzHugh, 2006), which was constructed from a damped van der Pol oscillator model (named the Bonhoeffer-van der Pol model by Fitzhugh). Indeed, the FHN model was introduced as a nerve model to simplify the Hodgkin-Huxley model (Hodgkin and Huxley, 1952a,b) and to facilitate analytic calculations:

(4) $$\begin{array}{l} \text{F}\left(U_{m},w_{m}\right)=\mu U_{m}\left(A-U_{m}\right)\left(U_{m}-B\right)-\gamma^{2}w_{m}, \end{array}$$

where μ, *A*, *B* and γ are real parameters. Note that *c*_*m*_/(*ABμ*) ≈ τ_*d*_ corresponds to a depolarization time scale, *A* and *B* are coefficients establishing the magnitude of the saturating “plateau” potential while the sign of *A* tells whether the portion of fiber is excitable (*A* > 0) or self-sustained oscillating (*A* < 0), and γ^2^ > 0 is the coupling rate between *U*_*m*_ and *w*_*m*_. The coupling to membrane currents is described by the following form of the function **G** in Equation (2):

(5) $$\begin{array}{l} \text{G}\left(U_{m},w_{m}\right)=\alpha^{2}U_{m}-\nu_{0}w_{m}+\lambda , \end{array}$$

where α^2^ > 0 (real positive) is an inverse inductance per unit length, λ is a leaking current parameter that will be put to zero for simplicity, and $\nu_{0}^{-1}$ a relaxation time scale for voltage gated channel inactivation. Since Equation (2) is a RLC circuit analog, it is straightforward to show that the saturating plateau potential lasts for a typical refractory period RP ≈ (αγ)^−1^, similarly to the damped van der Pol oscillator (Van der Pol and Van der Mark, 1928; Takashi, 2007).

It takes a time τ_*d*_ ∽ 1 to 10 ms to depolarize one cell membrane. Actually, τ_*d*_ acts as a cut-off time scale for continuous models as Equation (2). For a linear capacitance $c_{m}≲10^{-6}$ F/m, and a linear conductivity κ ≲ 10^−10^ Ω^−1^ × m, an upstroke (ion channels releasing the electric energy stored by conversion of chemical bond energy) is estimated to travel at a typical velocity $c∽\sqrt{\kappa {\left(c_{m}\tau_{d}\right)}^{-1}}≳0.1$ m/s. This is consistent with the conduction velocity measured in the atria under various conditions *c* ∽ 0.1 to 1 m/s (Zipes et al., 2017). The upstroke spans a typical membrane length of order *l*_*d*_ ≈ *cτ*_*d*_ ∽ 0.1 to 10 mm (Figure 1B), which acts as a spatial cut-off scale in such continuous models. Note that for very slow conduction velocity and rapid upstroke, the upstroke length scale can decrease down to the longitudinal size of one cell. Mathematically, a single boundary stimulus gives rise to a unique traveling upstroke: *U*_*m*_(*x, t*) = *U*_*m*_(*x* − *ct*). Immediately following depolarization, other voltage gated ion channels start contributing in a way as to set the membrane potential back to its original negative value. The resting polarized state is then restored back in a typical time of order τ_*r*_ ≳ 100 ms in the heart (Hille, 2001; Plonsey and Barr, 2007; Macfarlane et al., 2011). Time history dependencies of channel activation define the absolute refractory period *RP* of duration ≳ 100 ms in the heart.

A traveling pulse solution of the partial differential (Equation 2) indeed corresponds to a homoclinic cycle in the FHN model biasymptotic to the resting electric potential stable fixed point (Beuter et al., 2003; Izhikevich, 2007; Fenton and Cherry, 2008; Guckenheimer and Kuehn, 2009). Homoclinic cycles are triggered locally by the advancing foot of the depolarizing front or by an external stimulus. To reproduce the observed variety of physiological properties of an excitable myocardium, nonlinear modifications or extra membrane currents were added to the coupling functions **F** and **G** as for instance in Bär and Eiswirth (1993), Karma (1993), Panfilov and Hogeweg (1993), Fenton and Karma (1998), Fenton et al. (2002), Fenton and Cherry (2008), and Zipes et al. (2017). However, in their FHN type, Equations (2), (4), and (5) are quite adapted to simulate the homoclinic orbits underlying AP cycles and cardiac pulse trains. The dynamical complexity of the original FHN model has attracted a lot of mathematical and numerical attention (Glass and Mackey, 1987; Izhikevich, 2007). Under periodic stimulation, period doubling bifurcations were shown to precede a transition to deterministic chaos (Nolasco and Dahlen, 1968; Glass and Mackey, 1979, 1987; Guevara et al., 1981; Chialvo et al., 1990; Fenton and Cherry, 2008). Interestingly, along the line of the diffusely coupled Fitzhugh-Nagumo (Equations 2–5), the generation of higher-dimensional hyperchaotic spatio-temporal dynamics was suggested as a possible explanation of the dynamic transition to fibrillatory states in cardiac tissue (Baier and Müller, 2004).

### 2.2. Inter-cellular Ion Conduction and GJC Dynamics

The cardiac cell-cell contacts, where electrical signal conduction occurs, are found at intercalated discs located mostly at the narrow end of elongated cardiomyocytes. On their lateral side, the cardiomyocytes are ensheathed by cell-matrix contacts (Figure 1) with weaker electrical coupling. This organization favors a synchronized unidirectional propagation of electrical signals through serial strands of cardiomyocytes. When this synchronization is compromised, life-threatening arrhythmias can develop, and ultimately can become an obstacle for the regeneration of damaged heart tissues. The cardiac intercalated discs (IDs) must therefore be resilient to both mechanical and electrical disturbances to ensure a fast and reproducible propagation of the electrical signal that initiates contraction throughout the heart. ID includes three main structures: (i) the desmosome and (ii) the adherens junction (AJ) that provide the mechanical strength and continuity of the cell-cell contact, and that are both connected to the cytoskeleton, and (iii) the gap junction (GJ) which couples the cells electrically and metabolically. Other proteins which are not directly involved in the cell-cell contact also reside in the ID, including ion channels. The close contact and communication between cardiac myocytes is therefore essential for proper heart functioning as a syncytium, for both electrical and mechanical signal conductions. The GJCs (type I) which are found in cardiac muscles are organized in hexagonal arrays (connexins), with 8–9 center-to-center spacing, and overall thickness ~ 15-16 nm. Despite their negligible size, as compared to the myocyte length, these junctions were soon recognized as discontinuous conduction zones of the myocardium (Spach, 1983), and they were suspected to offer a low resistance to the transfer of electrical signals and more surprisingly to enhance this transfer nonlinearly (Cole et al., 1988).

Several numerical and theoretical attempts have tried to reconcile the difference of scales (from membrane and GJCs thickness to cell length) in a continuous or quasi-continuous treatment of cardiac fiber excitability (Diaz et al., 1983; Joyner et al., 1984; Cole et al., 1988; Keener, 1990; Bub et al., 2005; Pumir et al., 2005; Hand and Griffith, 2010; Hand and Peskin, 2010; Lin and Keener, 2010, 2013). At the heart tissue level, it appears at first legitimate to discard the GJC discontinuities, except maybe for radical instances of static or permanent alterations of GJCs due e.g., to connexin expression depletion (Cole et al., 1988) or gap junction blocker like α-glycerrhetinic acid (Bub et al., 2005) over large areas. As the longitudinal electric field generated by repeatedly passing APs drives the GJCs, we propose in this study to consider the spatio-temporal dynamics of the GJC conductances especially at cardiac intercalated discs where most GJCs reside. Indeed, we do not explicitly account for the discreteness of the GJCs spatially, considering instead an average behavior, but we introduce a nonlinear coupling term in the conduction current mono-domain cable (Equation 2) to mark the distinct propagation and polarization dynamics of GJCs and channel-gated ion channels.

Transjunctional voltage gated inactivation and recovery rates are comparable to repolarization rates (Neyton and Trautmann, 1986; Wang et al., 1992; Desplantez et al., 2007). This is a clue that we regard as pivotal for the behavior of ion transport along the direction of propagation at the locations of GJCs, especially during rapidly beating electrical activity (AP). As compared to previous AP propagation models involving GJCs (Burt and Sray, 1988; Kumar and Gilula, 1996; Harris, 2001; Bukauskas and Verselis, 2004; Danik et al., 2004; Perrachia, 2004), we put more attention on the dynamical implication of GJC (slow) kinetics in response to positive charge accumulation (capacitive charging). By considering that the capacitive charging of closed GJCs is proportional to the voltage drop across the interstitial space, we show that the GJC slow kinetics can enhance the effect thereby amplifying fluctuations around a stationary conductance. The whole AP propagation can therefore be strongly affected because small initial fluctuations of the GJC conductance may be amplified to non negligible values corresponding to a sort of nonlinear resonance of the GJC dynamics.

Averaging over typical depolarization time and length scales, corresponding to several thousands of intercalated discs, allows to derive a continuous mathematical model of inter-cellular ion condition and GJC dynamics. When combined with the well controlled FHN mono-domain mode, we get the following 1D system of nonlinearly coupled PDEs:

(6a,6b,6c and 6d) $$\begin{array}{l} \{\begin{array}{c} c_{m}\frac{\partial }{\partial t}U_{m}=\mu U_{m}\left(A-U_{m}\right)\left(U_{m}-B\right)\\ -\gamma^{2}w_{m}-c_{g}^{-1}\frac{\partial }{\partial x}\left(g\rho_{g}\right)+\kappa \frac{\partial^{2}}{\partial x^{2}}U_{m},\left(6\text{a}\right)\\ \frac{\partial }{\partial t}w_{m}=\alpha^{2}U_{m}-\nu_{0}w_{m}+\lambda ,\left(6\text{b}\right)\\ \frac{\partial }{\partial t}g=+\omega^{2}\rho_{g}-\nu_{1}g,\left(6\text{c}\right)\\ \frac{\partial }{\partial t}\rho_{g}=-g\frac{\partial }{\partial x}U_{m}-\nu_{2}\rho_{g},\left(6\text{d}\right) \end{array} \end{array}$$

with the same boundary conditions as defined in Equation (3). The new dynamical variables are: (i) the GJC conductance fluctuations *g* (around its mean value accounted for in κ) (in *S* ≡ Ω^−1^), which can be interpreted as the non zero gradient part $g\equiv \frac{\partial }{\partial x}\kappa \neq 0$, and (ii) the average capacitive charge linear density ρ_*g*_. The new parameters are *c*_*g*_: an average linear capacitance analog to *c*_*m*_ but for the intercalated disks, ω^2^: a reaction rate for the zeroth-order kinetic Equation (6c), and ν_1_ a relaxation rate (ν_2_ → 0). Note that the zeroth-order (independent of *g*) reaction rate is justified because only a small fraction of abnormally closing GJCs have to grow from zero initially, while a majority of others remain conducting. At the same time, capacitive charges act on all nearby hexamers. In addition, current conservation modifies Equation (2) so as to include a current term coming from the capacitive charging of GJCs. Precisely, the local ohmic current flowing through open GJCs experiences losses due to closed GJCs. Thus, adding the GJC current as $I_{g}=c_{g}^{-1}g\rho_{g}$, to the unpertubed conduction current $I_{\Omega }=-\kappa \frac{\partial }{\partial x}U_{m}$, gives a total longitudinal current *I* = *I*_Ω_ + *I*_*g*_ (Figure 1B). From charge conservation hypothesis, taking the divergence of *I* yields Equation (6a). Equation (6d) is obtained by considering that the net capacitive charging current density $\frac{\partial }{\partial t}\rho_{g}$ is non zero as soon as the GJCs start closing massively. This current density is written as the product of the GJC conductance and the local electric field produced by the AP wave ($-\frac{\partial }{\partial x}U_{m}$). Finally, ν_1_ accounts for the GJC relaxation rate while ν_2_ (with ν_2_ ≪ ν_1_) accounts for other charge leakages. We refer the reader to Table 1 for more details on units and numerical values.

**Table 1:** Parameters used in the simulations of Equations (6)–(8).

**Param**	**L**	***c*_*m*_**	***c***_*g*_	**κ**	**μ**	**A**	**B**	**ν**_**0**_	**ν**_**1**_	**ν**_**2**_	**γ**^**2**^	**α**^**2**^	**ω**^**2**^	**λ**
Simul #1	210	1	1	0.01	3	0.1	1	0.02	0.01	0.0001	3	0.008	10^5^	0
Simul #2	150	1	1	0.01	3	0.1	1	0.02	0.01	0.0001	3	0.008	10^5^	0
Simul #3	90	1	1	0.01	3	0.1	1	0.02	0.01	0.0001	3	0.008	10^5^	0
Simul #4	30	1	1	0.01	3	0.1	1	0.02	0.01	0.0001	3	0.008	10^5^	0
Simul #5	150	1	1	0.005	6	0.1	1	0.01	0.005	0.0001	6	0.004	5.10^4^	0
Simul #6	150	1	1	0.04	3	0.1	1	0.02	0.01	0.0001	3	0.008	10^5^	0
Simul #7	150	1	1	0.08	3	0.1	1	0.02	0.01	0.0001	3	0.008	10^5^	0

*The spatial size is L in δx = 0.3 mm units (section 2.3). The parameter dimensions are: c_m_ (ms × mm^−1^ × Ω^−1^), c_g_ (ms × mm^−1^ × Ω^−1^), κ (mm × Ω^−1^), ω^2^ (mm × ms^−2^ × mV^−1^), ν_i_ (ms^−1^) for 0 ≤ i ≤ 2; α^2^ (ms^−2^), γ (no unit), μ (mm^−1^ × Ω^−1^ × mV^−2^), A, B (mV)*.

The boundary conditions are defined such that the value of *A* is chosen opposite (*A* → −*A*) for *x* = 0, making this fiber end self-sustained oscillating, and unconstrained for *x* = *L* with null electric field $-\frac{\partial }{\partial x}U_{m}\left(x=L\right)=0$, while no external current is added *I*_*ext*_ = 0. Thus, we set :

(7) $$\begin{array}{l} c_{m}\frac{\partial }{\partial t}U_{m}=\mu U_{m}\left(-A-U_{m}\right)\left(U_{m}-B\right)-\gamma^{2}w_{m}\\ -c_{g}^{-1}g\rho_{g}\text{ for }x=0, \end{array}$$

and

(8) $$\begin{array}{l} \frac{\partial }{\partial t}\rho_{g}=-\nu_{2}\rho_{g}\text{ for }x=L. \end{array}$$

### 2.3. Numerical Scheme

We use standard finite difference techniques to integrate numerically the system of partial differential Equation (6) with boundary conditions defined in Equations (7) and (8). The linear Laplacian operator is handled with the standard “Crank-Nicholson” scheme using tri-diagonalization. But, one notable unusual trait, as compared to other cardiac models, is that by construction here, the spatial operator is no longer elliptic because of the divergence term. This calls for a special treatment of the gap junction fluctuating quantities.

One way to find out a stable scheme is by considering the following heuristic. For the sake of exposition simplicity, the mean membrane potential is assumed adiabatically constant $\frac{\partial }{\partial t}U_{m}=0$. Equation (6d) possesses an important mirror antisymmetry upon changing *U*_*m*_ → −*U*_*m*_ and *x* → −*x*, which distinguishes flow directions of GJ charging or discharging, for a given fluctuating conductance. Consistently, Equations (6b, c) are invariant under the change ρ_*g*_ → −ρ_*g*_ and *g* → −*g*. Hence, the fluctuating vector $W=\left[\begin{array}{l} \rho_{g}\\ g \end{array}\right]$ obeys the following temporal evolution equation:

(9) $$\begin{array}{l} \frac{\partial }{\partial t}W=\text{M}W, \end{array}$$

where the matrix local evolution operator is

(10) $$\begin{array}{l} \text{M}=\left[\begin{array}{cc} -\nu_{2} & -\frac{\partial }{\partial x}U_{m}\\ \omega^{2} & -\nu_{1} \end{array}\right], \end{array}$$

which shows (ν_2_ → 0) that the quantity $\theta =\omega \sqrt{\left|\frac{\partial }{\partial x}U_{m}\right|}$ can act, for some fluctuation, as a typical growth rate (as in the case of a right moving depolarizing front for which the gradient is negative) or as a typical pulsation frequency otherwise. To handle this particular linear, (temporal) evolution, the so-called “leap-frog” method is usually well adapted and was found numerically stable.

When fluctuations can be assumed as happening over a local spatial distance δℓ such that a positive variation from zero is $W≃-\delta \ell \frac{\partial }{\partial x}W$, one can rewrite dimensionally the temporal evolution operator (Equation 10) into a spatial transfer operator: $\text{M}W\to -{\text{M}}^{\prime }\frac{\partial }{\partial x}W-\nu W$ where

(11) $$\begin{array}{l} {\text{M}}^{\prime }=\left[\begin{array}{cc} 0 & \delta \ell \frac{\partial }{\partial x}U_{m}\\ -\delta \ell \omega^{2} & 0 \end{array}\right], \end{array}$$

and $v=\left[\begin{array}{l} v_{2}\\ v_{1} \end{array}\right]$. Thus, an advection velocity appears of magnitude *v* = δ*ℓθ*, directed in one or the other way depending on the sign of the fluctuating variables. If not further driven, these fluctuating variables are damped over a spatial distance (along a characteristic) Δ*x* = ±*vΔt*, in a time $\Delta t\sim \nu_{1}^{-1}$. A standard “upwind” scheme takes good care of the numerical stability of advection, with sufficient precision, but favors a particular direction of propagation for positive fluctuations, here in agreement with the mirror antisymmetry. This also explains why we choose to place on the outer left side of our 1D system a rapidly beating cell (Equation 7) mimicking an ectopic source, whereas the dynamics is monitored only on its right, at different spatial positions. The outer right boundary is chosen non-excitable in the present study, which mimics any kind of connection to non-conducting tissues. Numerical integration values of time step δ*t* = 0.4 ms for all simulations in Table 1 (except Simul #5 for which δ*t* = 0.25 ms), and spatial step δ*x* = 0.3 mm are chosen to abide by the Courant-Friedrichs-Lewy condition *vδt*/δ*x* < 1, for which an upper bound estimate of the conduction velocity is *c* ≃ 0.1 m/s.

### 2.4. Software and Documentation

The numerical integration code can be downloaded directly from the following link: https://geostat.bordeaux.inria.fr/images/fwd-matlab-code.zip or through Geostat team software page: https://geostat.bordeaux.inria.fr/index.php/downloads.html (last item).

### 2.5. From the Formal Model Variables to Experimentally Recorded Potential Values

**Figure 2:**
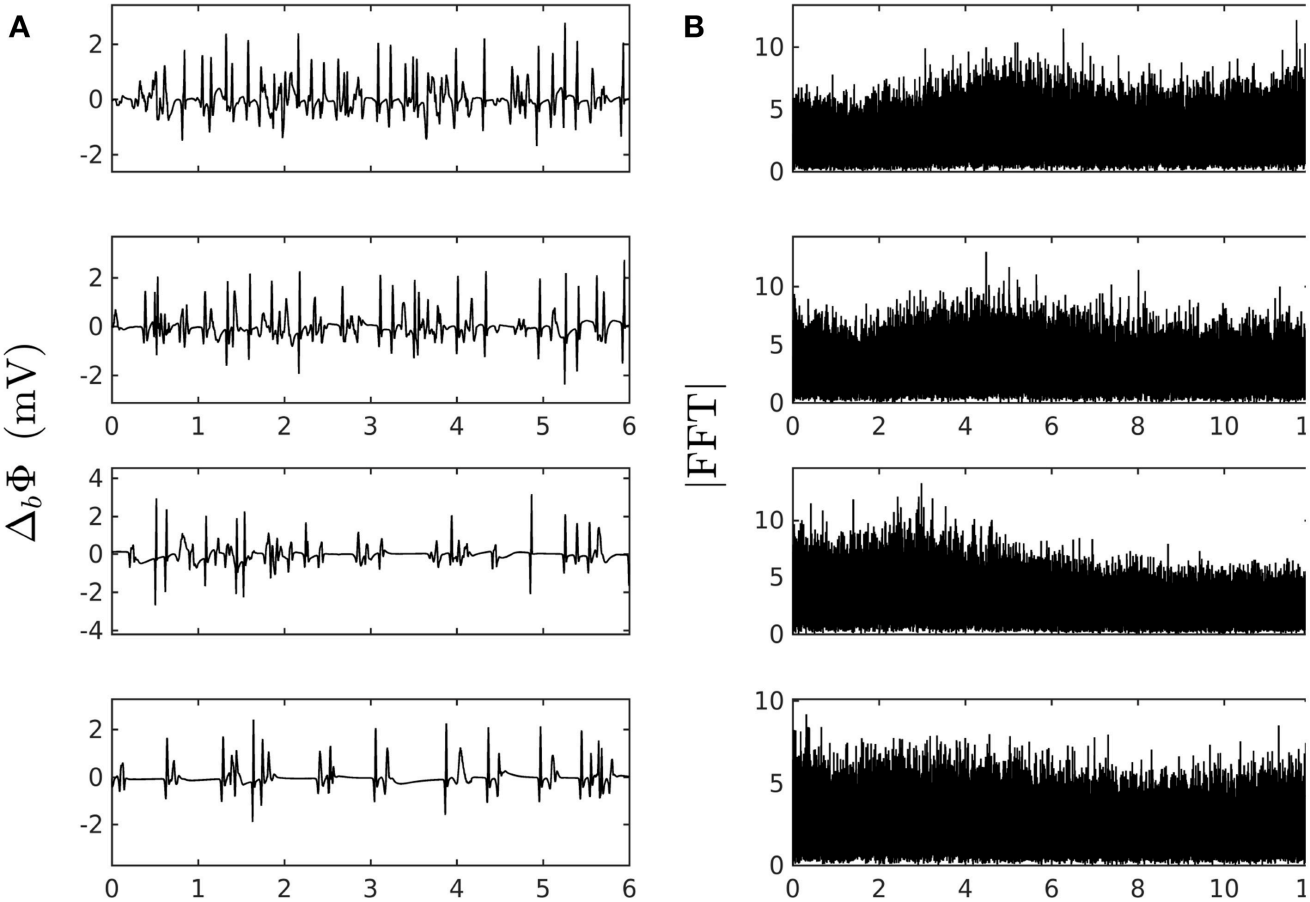
Numerically simulated pseudo bipolar potential. **(A)** 6 s time-series Δ_*b*_ Φ (Equation 12) are generated at positions *x* (in δ*x* = 0.3 mm units) = 8, 18, 75 and 134 (from top to bottom) with our model defined in Equations (6)–(8) with parameter values defined in Simul #2 (Table 1), *L* = 150 (in δ*x* = 0.3 mm units). **(B)** Corresponding Fourier transform power spectra. No attempt was made to reproduce the high and low pass filtering used in real bipolar catheter acquisitions. The natural point source frequency at the boundary is ∽ α γ ≈ 5 Hz. Rarefaction of pulses occurs as one moves away from the source which is a hint at some randomly back-scattered pulses that collide and annihilate up-following pulses without reexciting new pulses.

We simulated the external electric potential recorded by a bipolar electrode Δ_*b*_ Φ by use of the dipole layer approximation. Within the mono-domain framework, the electric potential felt just outside a cardiac fiber of thickness *e* ∽ 2 mm is evaluated as (Plonsey and Barr, 2007; Macfarlane et al., 2011):

(12) $$\begin{array}{l} \Delta_{b}\Phi \left(x,t\right)=\Pi_{p}⋆i_{d}\left(x,t\right)=\int \Pi_{p}\left(x-x^{\prime };e\right)\\ \left(\kappa \frac{\partial^{2}}{\partial x^{2}}U_{m}\left(x^{\prime },t\right)-i_{g}\left(x^{\prime },t\right)\right)\text{d}x^{\prime }, \end{array}$$

where Π_*p*_ is the convolution function for the bipolar probe, $i_{d}\left(x,t\right)=\kappa \frac{\partial^{2}}{\partial x^{2}}U_{m}\left(x,t\right)-i_{g}\left(x,t\right)$ the total dipole layer current divergence (or flux across a fiber section), and $i_{g}\left(x,t\right)=\frac{\partial }{\partial x}I_{g}\left(x,t\right)$ the new contribution coming from GJC losses. Since we are interested in variations over distances greater than the depolarization length scale, a good approximation is to compute $\Pi_{p}\left(x\right)=\frac{\partial }{\partial x}\Pi \left(x,e\right)$, where $\Pi \left(x,e\right)=2\pi \left(\frac{x}{\left|x\right|}-\frac{x}{\sqrt{x^{2}+e^{2}}}\right)\approx \int |r{|}^{-3}\vec{r}\cdot \vec{\text{d}S}$ is the solid angle spanning the fiber section at a distance $\vec{r}=\left(x,e\right)$ of the depolarization front from the probe. Figure 2 shows typical pseudo bipolar potential time series numerically simulated with our 1D system of PDEs (Equation 6) with, as boundary condition at *x* = 0 (Equation 7), an automatically beating source of frequency αγ ~ 5 Hz so as to match the cardiac pulse trains observed experimentally. Tuning the newly introduced parameters ω^2^ and ν_1_ in Equation (6), we have found quite easily paths leading from a phase of coherent propagation of AP pulses to a phase of quite incoherent and intermittent electrical activity (Figure 2) that strongly reminds the very irregular behavior of electric potential time series recorded during AF (see for comparison Figure 1 in our companion paper I Attuel et al., 2017). Besides the obvious interest of analyzing the succession of bifurcations and transition events encountered along these paths in parameter space, we will focus in this paper on a comparative study of the complex and highly intermittent modulation of cardiac pulse trains simulated numerically with our model of cardiac AP conduction and GJC dynamics and the one observed experimentally in the coronary sinus during episodes of AF (Attuel et al., 2017).

## 3. Methods of Analysis

### 3.1. Local Impulse Energy

With the same convention as in the companion paper I (Attuel et al., 2017), the local 1D impulse electric energy traveling with scarcely any alteration at velocity *c* over a depolarization time period τ_*d*_, is evaluated from Equation (12) as:

(13) $$\begin{array}{l} E\left(x,t\right)={\left(\frac{\partial \Delta \phi_{b}\left(x,t\right)}{\partial t}\right)}^{2}, \end{array}$$

after dropping some constant prefactors. To practically derive *E*(*x, t*) from the numerically simulated Δ_*b*_ ϕ (*x, t*), we used the same order 4 finite difference scheme as in the companion paper I (Attuel et al., 2017).

### 3.2. Zooming on the Local Impulse Energy With the Wavelet Transform Microscope

**Figure 3:**
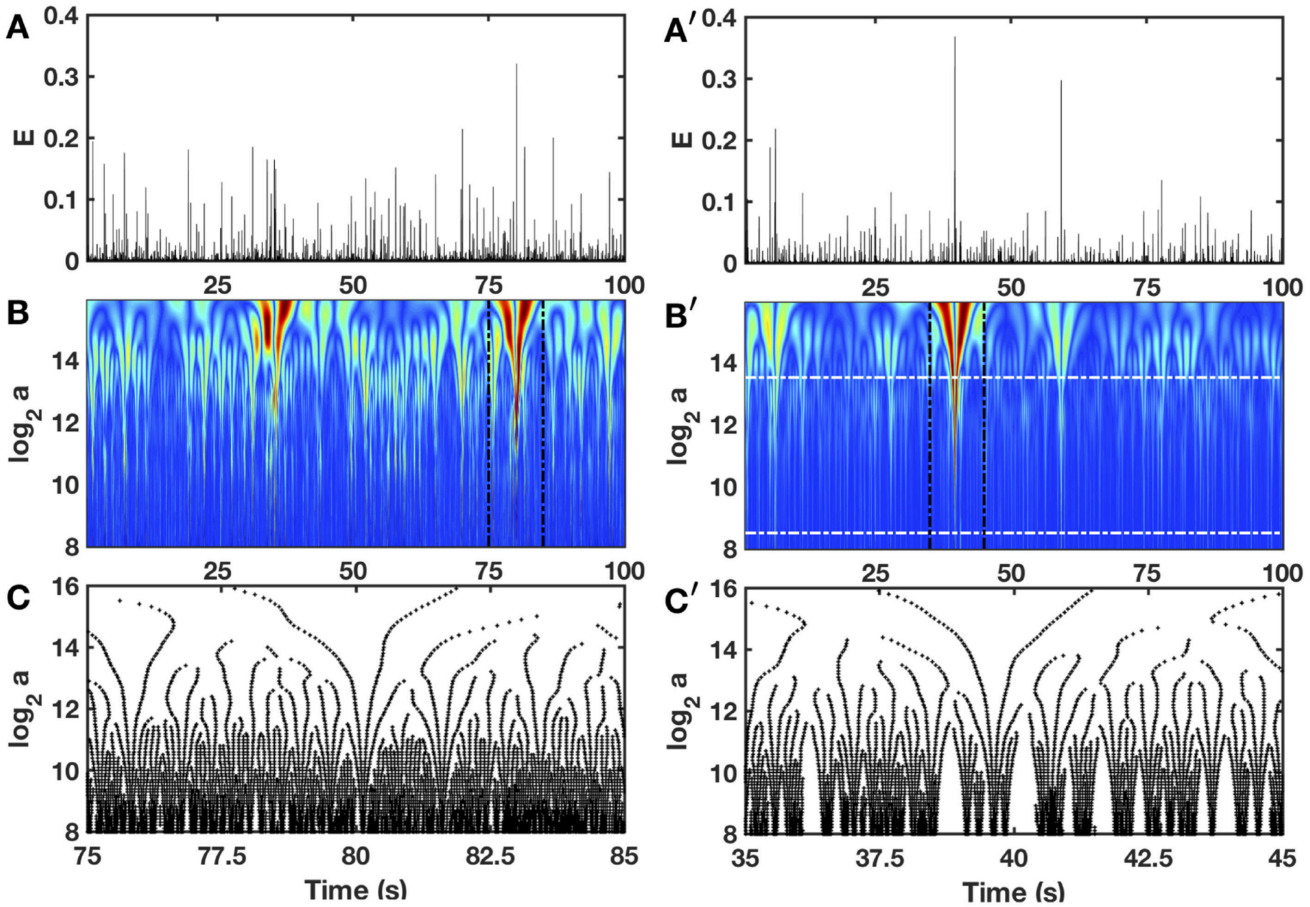
Wavelet transform of local impulse energy time-series. **(A)** A 100 s portion of *E*(*t*) recorded at the electrode Pt3 (Companion paper I Attuel et al., 2017). **(B)** Time-scale WT representation of *E*(*t*) with the third-order analyzing wavelet *g*^(3)^ (Equation 16). The modulus of the WT is coded, independently at each scale *a*, using 256 colors from black ($|T_{g^{\left(3\right)}}\left(t,a\right)|=0$) to red ($\max_{t}|T_{g^{\left(3\right)}}\left(t,a\right)|$). **(C)** WT skeleton defined by the maxima lines of a 10 s portion of *E*(*t*). The scale *a* = α*Δt*/δ*t*, where α is an analyzing wavelet dependent constant (α = 8.6 10^−2^ for *g*^(3)^ with the lastwave software), and δ*t* = 0.4 ms. **(A′–C′)** same as **(A–C)** for a numerical time series *E*(*x, t*) generated at position *x* = 75 (in δ*x* = 0.3 mm units) with our model defined in Equations (6)–(8) with the set of parameter values used in Simul #2 (Table 1), and a total system length *L* = 150 (in δ*x* = 0.3 mm units). In **(B′)** the white horizontal dashed-dotted lines delimit the range of time scales (2^8.5^ ≤ *a* ≤ 2^13.5^) used to perform linear regression fit estimates of the τ(*q*) and *D*(*h*) multifractal spectra.

In Figure 3 is shown in a comparative time-scale decomposition of two 100 s long local (*x* fixed) impulse energy time-series, the first one was experimentally recorded at the electrode Pt3 in a patient with chronic AF (companion paper I, Attuel et al., 2017) and the second ones was numerically simulated with Equations (6)–(8) with model parameters defined in Simul #2 (Table 1). The continuous wavelet transform (WT) consists in expanding signals in terms of wavelet constructed from a single function, the “analyzing wavelet” ψ, by means of translations and dilations (Grossmann and Morlet, 1984; Daubechies, 1992; Meyer, 1992; Mallat, 1998):

(14) $$\begin{array}{l} T_{\psi }\left[E\right]\left(t_{0},a\right)=\frac{1}{a}\int_{-\infty }^{+\infty }E\left(t\right)\psi \left(\frac{t-t_{0}}{a}\right)dt, \end{array}$$

where *t*_0_ is a time parameter and *a* (> 0) a scale parameter (inverse of frequency). Interestingly, by choosing a wavelet ψ which has its first *n*_ψ_ moments null $\left[\int t^{m}\psi \left(t\right)dt=0,0\leq m<n_{\psi }\right]$, it can be proven (Arneodo et al., 1988, 1995b, 2008; Jaffard, 1989; Muzy et al., 1991, 1994; Mallat and Hwang, 1992) that:

(15) $$\begin{array}{l} T_{\psi }\left[E\right]\left(t_{0},a\right)∽a^{h\left(t_{0}\right)},a\to 0^{+}, \end{array}$$

provided *n*_ψ_ > *h*(*t*_0_), where *h*(*t*_0_) is the point-wise Hölder exponent that characterizes the maximum regularity of the signal *E* at point *t*_0_. As experienced in the companion paper I (Attuel et al., 2017) for experimental local impulse energy time-series, we will use in this work the third derivative of a Gaussian function as analyzing wavelet with *n*_ψ_ = 3 (Muzy et al., 1994; Arneodo et al., 1995b) (Figure S1 in the companion paper I):

(16) $$\begin{array}{l} g^{\left(3\right)}\left(t\right)=\frac{d^{3}}{dt^{3}}\left(e^{-t^{2}/2}\right). \end{array}$$

Interestingly, to track cusp singularities (for oscillating singularities like chirps see Arneodo et al., 1995a, 1997), the WT skeleton defined by the so-called maxima lines of local WT modulus maxima (WTMM) (Figures 3C,C'), was proved to be of practical use since along these maxima lines, Equation (15) was shown to apply (Mallat and Hwang, 1992).

### 3.3. A Wavelet-Based Multifractal Formalism

The wavelet transform modulus maxima (WTMM) method (Muzy et al., 1991, 1994; Bacry et al., 1993; Arneodo et al., 1995b, 2008) was originally developed to generalize box-counting techniques (Arneodo et al., 1987) and to remedy the limitations of the structure functions method (Muzy et al., 1993) in performing multifractal analysis of one-dimensional (1D) velocity signals in fully-developed turbulence. It has proved very efficient to estimate scaling exponents and multifractal spectra (Muzy et al., 1994; Audit et al., 2002; Arneodo et al., 2008; Ivanov et al., 2009). The WTMM method has been extensively applied in different domains of fundamental and applied science, including the analysis of complex time-series found in genomics (Nicolay et al., 2007; Arneodo et al., 2011; Audit et al., 2013) and physiological systems (Ivanov et al., 1999, 2001, 2009; Nunes Amaral et al., 2001; Goldberger et al., 2002; Ciuciu et al., 2012; Chudácek et al., 2014; Gerasimova et al., 2014; Richard et al., 2015; Gerasimova-Chechkina et al., 2016). The WTMM method and its wavelet leaders discrete generalization (Jaffard et al., 2007; Wendt et al., 2007) have already been applied to cardiac signals but mainly to characterize the fluctuations of inter-beat intervals (Ivanov et al., 1999, 2001; West et al., 2004; Leonarduzzi et al., 2010; Wendt et al., 2014; Gadhoumi et al., 2018). As for the analysis of experimental local impulse energy time-series in the companion paper I (Attuel et al., 2017), we will use here two declinations of the WTMM method, the moment (partition function) method and the magnitude cumulant method (Muzy et al., 1994; Arneodo et al., 2008).

The method of moments consists in investigating the scaling behavior of partition functions defined in term of WTMM:

(17) $$\begin{array}{l} Z\left(q,a\right)=\underset{l\in L\left(a\right)}{\sum }|T_{\psi }\left[E\right]\left(t,a\right){|}^{q}∽a^{\tau \left(q\right)},a\to 0^{+}, \end{array}$$

where *q* ∈ ℝ, L(a) is the set of the maxima lines *l* that defines the WT skeleton and the exponents *q* and τ(*q*) play, respectively, the role of an inverse temperature and a free energy in the analogy that links the multifractal formalism and thermodynamics (Bohr and Tél, 1988; Arneodo et al., 1995b). Then, from the Legendre transform of τ(*q*):

(18) $$\begin{array}{l} D\left(h\right)=\underset{q}{\min}\left[qh-\tau \left(q\right)\right], \end{array}$$

we get as equivalent of entropy, the so-called *D*(*h*) singularity spectrum defined as the fractal (Hausdorff) dimension of the set of points *t* where the Hölder exponent (equivalent of energy) *h*(*t*) = *h* (Bacry et al., 1993; Muzy et al., 1993, 1994; Arneodo et al., 1995b; Jaffard, 1997a,b).

In practice, to avoid instabilities in performing the Legendre transform, we instead compute the following expectation values (Muzy et al., 1994; Arneodo et al., 1995b), analogous to the fundamental thermodynamic relations, by inversion of Equation (18):

(19) $$\begin{array}{l} h\left(q,a\right)=\frac{\partial }{\partial q}\ln\left(Z\left(q,a\right)\right)=\underset{l\in L\left(a\right)}{\sum }\ln\;\left(\left|T_{\psi }\left[E\right]\left(l,a\right)\right|\right)\cdot W_{\psi }\left[E\right]\left(q,l,a\right), \end{array}$$

and

(20) $$\begin{array}{l} D\left(q,a\right)=q\frac{\partial }{\partial q}\ln\left(Z\left(q,a\right)\right)-\ln\left(Z\left(q,a\right)\right)\\ \;=\underset{l\in L\left(a\right)}{\sum }W_{\psi }\left[E\right]\left(q,l,a\right)\cdot \ln\;\left(W_{\psi }\left[E\right]\left(q,l,a\right)\right), \end{array}$$

where $W_{\psi }\left[E\right]\left(q,l,a\right)=|T_{\psi }\left[E\right]\left(l,a\right){|}^{q}/Z\left(q,a\right)$ corresponds to the Bolzmann weight (Arneodo et al., 1995b). Then, from the slopes of *h*(*q, a*) and *D*(*q, a*) vs. ln *a*, we get *h*(*q*) and *D*(*q*), and therefore the *D*(*h*) singularity spectrum as a curve parametrized by *q*.

An alternative method that can be used as a double check of the predictions of the method of moments is the so-called method of magnitude cumulants (Delour et al., 2001). This method consists in computing the cumulants *C*_*n*_(*a*) of the magnitude ln |*T*_*a*_|. Then from the behavior of the cumulants:

(21) $$\begin{array}{l} \begin{array}{c} C_{1}\left(a\right)\equiv \langle \ln\;|T_{a}|\rangle ∽c_{1}\ln\;\left(a\right),\\ C_{2}\left(a\right)\equiv \langle \ln^{2}|T_{a}|\rangle -{\langle \ln\;|T_{a}|\rangle }^{2}∽-c_{2}\ln\;a,\\ C_{3}\left(a\right)\equiv \langle \ln^{3}|T_{a}|\rangle -3\langle \ln^{2}|T_{a}|\rangle +2{\langle \ln\;|T_{a}|\rangle }^{3}∽c_{3}\ln\;a,\\ \cdot \cdot \cdot \end{array} \end{array}$$

we get the following expansion formula for τ(*q*):

(22) $$\begin{array}{l} \tau \left(q\right)-c_{0}+c_{1}q-c_{2}q^{2}/2!+c_{3}q^{3}/3!\cdot \cdot \cdot \end{array}$$

where the coefficients *c*_*n*_ > 0 are estimated as the slope of *C*_*n*_(*a*) vs. ln *a* (*n* = 1, 2, 3,, ··· ), and *c*_0_ = *D*_*F*_ as the fractal dimension of the support of singularities of *E*(*t*).

Multifractal analysis allows us to distinguish monofractal signals of unique Hölder exponent *H*. Their τ(*q*) spectrum is a linear function of *q* with slope *c*_1_ = *H*, all the other *c*_*i*_ = 0, *i* ≥ 2. The corresponding *D*(*h*) singularity spectrum reduces to a single point *D*(*h* = *c*_1_) = *D*_*F*_. In contrast, a nonlinear τ(*q*) is the signature of multifractal signals with Hölder exponent *h*(*t*) fluctuating over time (Muzy et al., 1991, 1994; Bacry et al., 1993; Arneodo et al., 1995b, 2002, 2008). As in the companion paper I (Attuel et al., 2017), in this study we will fit the numerical data by so-called log-normal quadratic approximation

(23) $$\begin{array}{l} \tau \left(q\right)=-c_{0}+c_{1}q-c_{2}q^{2}/2, \end{array}$$

leading to a quadratic single hump shaped *D*(*h*) singularity spectrum

(24) $$\begin{array}{l} D\left(h\right)=c_{0}-{\left(h-c_{1}\right)}^{2}/2c_{2}, \end{array}$$

where *c*_0_ = −τ(0) = *D*_*f*_ is the fractal dimension of the support of singularities of *E*(*t*), *c*_1_ is the value of *h* that maximizes *D*(*h*), and the intermittency coefficient *c*_2_ characterizes the width of the *D*(*h*) spectrum (Delour et al., 2001).

### 3.4. Beyond One-Point Statistics: The Two-Point Magnitude Correlation Method

Many studies have misleadingly extrapolated a multifractal diagnosis to the existence of an underlying multiplicative cascade process. To address this issue, we indeed need to investigate two-point statistics. The two-point magnitude correlation method amounts to investigate how the two-point magnitude correlation function (Arneodo et al., 1998a)

(25) $$\begin{array}{l} C(a,\Delta t)\;=\;\langle \left(\ln|T_{a}(t)|-\langle \ln|T_{a}(t)|\rangle \right)\cdot \\ \;\left(\ln|T_{a}(t+\Delta t)|-\langle \ln|T_{a}(t)|\rangle \right)\rangle \end{array}$$

changes as a function of Δ*t* at scale *a*. As demonstrated by Arneodo et al. (1998a,b) for random multiplicative cascades on wavelet dyadic trees (see also Meneveau and Sreenivasan, 1991):

(26) $$\begin{array}{l} C\left(a,\Delta t\right)∽-c_{2}\ln\;\Delta t,\Delta t>a, \end{array}$$

where the proportionality coefficient *c*_2_ is the intermittency coefficient defined in Equations (21) and (22) [Note that *C*(*a*, Δ*t* = 0) ≡ *C*_2_(*a*) ∽ − *c*_2_ln *a*]. Thus, by computing *C*(*a*, Δ*t*) from Equation (25) and plotting it as a function of ln Δ*t*, inferences can be made about long-range dependence and consistency with a multiplicative cascading process (Arneodo et al., 1998a,b). Applications of the two-point magnitude correlation method have already provided insight into a wide variety of problems, e.g., the validation of the log-normal cascade phenomenology of fully developed turbulence (Arneodo et al., 1998a,c, 1999) and of high resolution temporal rainfall (Venugopal et al., 2006; Roux et al., 2009), and the demonstration of the existence of a causal cascade of information from large to small scales in financial time series (Arneodo et al., 1998d; Muzy et al., 2001). In the companion paper I (Attuel et al., 2017), we have applied this method to experimental local impulse energy time-series during episodes of AF. Surprisingly, this study has revealed the absence of an underlying multiplicative time-scale structure that will be used in this work as a multifractal random noise numerical test of the pertinence of our cardiac excitable network modeling (section 2) during AF.

## 4. Results

### 4.1. One-Point Multi-Fractal Analysis of Local Impulse Energy Numerical Data

#### 4.1.1. Multifractal Analysis of the Simulated Impulse Energy Time-Series With the WTMM Method of Moments

**Figure 4:**
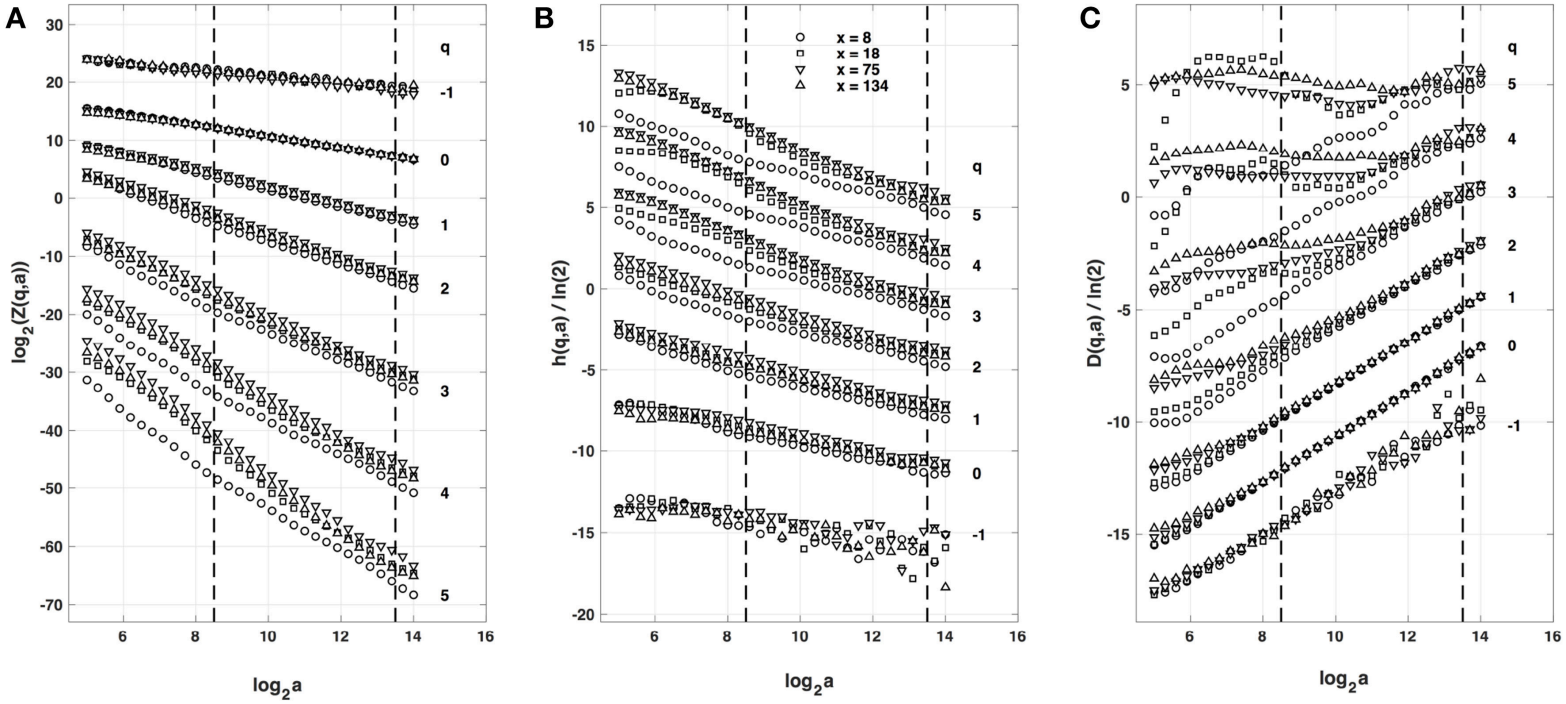
Multifractal analysis of local impulse energy time-series *E*(*x, t*) generated with our model defined in Equations (6)–(8) with the parameter values defined in Simul #2 (Table 1), *L* = 150 (in δ*x* = 0.3 mm units). **(A)** log_2_*Z*(*q, a*) vs. log_2_*a* (Equation 17). **(B)** *h*(*q, a*)/ln 2 *vs* log_2_*a* (Equation 19). **(C)** *D*(*q, a*)/ln 2 vs. log_2_*a* (Equation 20). The computation were performed with the WTMM method (Paper I, Methods of Analysis Attuel et al., 2017) for different values from *q* = −1 to 5 with the analyzing wavelet *g*^(3)^ (Equation 16). The vertical dashed lines delimit the range of scales (2^8.5^ ≤ *a* ≤ 2^13.5^) used for the linear regression estimate of τ(*q*), *h*(*q*) and *D*(*q*) in Figure 5. The symbols correspond to the time-series *E*(*x, t*) computed at the spatial positions *x* = 8 (◦), 18 (□), 75 (▽) and 134 (△) (in δ*x* = 0.3 mm units).

**Figure 5:**
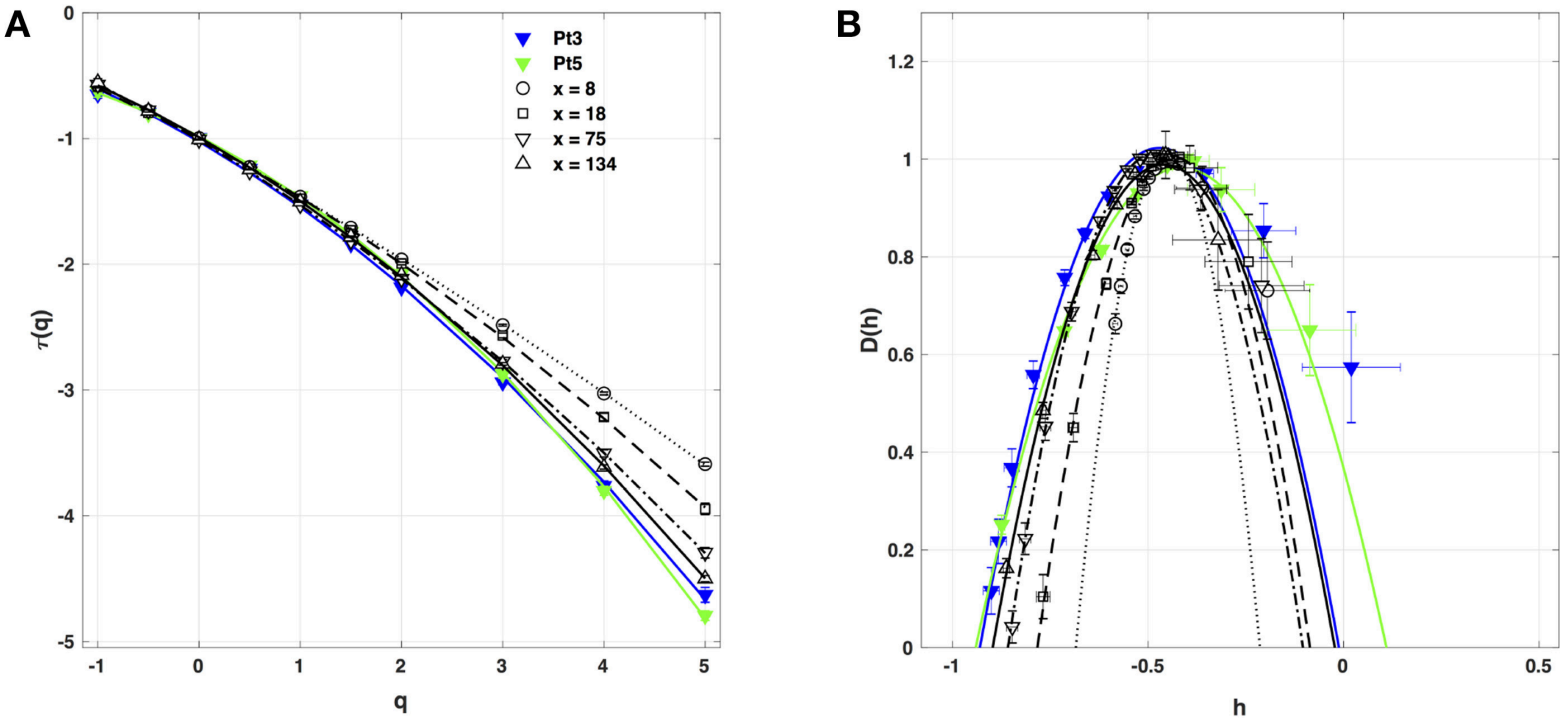
Multifractal spectra of local impulse energy time-series *E*(*x, t*) generated with our model defined in Equations (6)–(8) with the set of parameter values defined in Simul #2 (Table 1), *L* = 150 (in δ*x* = 0.3 mm units). **(A)** τ(*q*) vs. *q* estimated by linear regression fit of log_2_*Z*(*q, a*) vs. log_2_*a* (Figure 4A). **(B)** *D*(*h*) vs. *h* obtained from linear regression fits of *h*(*q, a*) (Figure 4B) and *D*(*q, a*) (Figure 4C) vs. log_2_*a*. The symbols have the same meaning as in Figure 4. The curves correspond to quadratic spectra Equations (23) and (24) with parameters [*c*_0_, *c*_1_, *c*_2_] reported in Table 2 for time-series *E*(*x, t*) computed at the spatial positions *x* = 8 (◦), 18 (□), 75 (▽) and 134 (△) (in δ*x* = 0.3 mm units). For comparison are reported the spectra previously obtained for the experimental time-series recorded at the electrodes Pt3 (blue ▼) and Pt5 (green ▼) in the left atrial posterior wall (Companion paper I Attuel et al., 2017).

When applying the WTMM method to the impulse energy time-series *E*(*x, t*) obtained by numerically integrating Equation 6 with periodically driven boundary condition (Equation 7) at *x* = 0 (Figure 3A'), we confirmed that the partition function *Z*(*q, a*) (Equation 17) obtained from the WT computed with the analysing wavelet *g*^(3)^ (Figure 3B') and its skeleton (Figure 3C'), displays scaling properties for *q* = −1 up to ≲ 5 over a range of time-scales larger than the typical interbeat ~ 0.2 s (Figure 2B). We strictly limited this range to (1.7, 54 s) for linear regression fit estimates in a logarithmic representation (Figure 4A). The τ(*q*) spectrum so-obtained at different spatial positions *x* = 8, 18, 75 and 134 for a 1D system of total length *L* = 150 (*x* and *L* expressed in δ*x* = 0.3 mm units) is robustly well approximated by a quadratic spectrum (Equation 23) with parameter values that do not change much when moving away from the periodic beating source at *x* = 0 (Table 2). The support of singularities of *E*(*x, t*) has a fractal dimension *c*_0_ = *D*_*F*_ ~ 1, independently of *x*. The singularities of Hölder *h* = *c*_1_ ~ 0.45−0.48 that maximizes *D*(*h*) are also consistently observed all along our 1D spatial system. Interestingly, the intermittency coefficient *c*_2_ is significantly different from zero (the hallmark of multifractal signals) and increases from values *c*_2_ ~ 0.03 close to the source up to values *c*_2_ ~ 0.1 far away from the source. Overall the values of *c*_0_, *c*_1_ and *c*_2_ parameters reported in Table 2 are very similar to the ones obtained in Table 1 of the companion paper I (Attuel et al., 2017) for electrodes Pt3 and Pt5 located in the left atrial posterior wall. Our numerical simulations thus reveals some increased intermittency of *E*(*x, t*), when moving away from the periodic beating source, rather rapidly converging to a τ(*q*) spectrum in quantitative agreement with the one observed experimentally (Figure 5A). This is confirmed when, respectively, plotting *h*(*q, a*)/ln 2 (Equation 19) and *D*(*q, a*)/ln 2 (Equation 20) vs. log_2_*a* (Figures 4B,C). Despite some deterioration of scaling for large *q* ≳ 4 values, from the estimate of the slopes *h*(*q*) and *D*(*q*), we get the single humped *D*(*h*) spectra shown in Figure 5B and which clearly widen when moving away from the source to ultimately match the *D*(*h*) spectra observed experimentally (Figure 5B). As a check of the reliability of the so-obtained *D*(*h*) spectra, they are found quite well approximated by the quadratic spectra defined in Equation 24 with the parameter values obtained from a polynomial fitting of the τ(*q*) data (Table 2, Figure 5).

**Table 2:** Results of the WTMM multifractal analysis of local impulse energy time-series *E*(*x, t*) numerically simulated with our model defined in Equations (6)–(8) with the set of parameter values defined in Simul #2 (Table 1), *L* = 150 (in δ*x* = 0.3 mm units).

	***x*** **= 8**	***x*** **= 18**	***x*** **= 75**	***x*** **= 134**
*c*_0_	1.004 ± 0.001	1.005 ± 0.001	1.013 ± 0.001	0.989 ± 0.002
*c*_1_	−0.449 ± 0.003	−0.435 ± 0.005	−0.481 ± 0.002	−0.460 ± 0.007
c*_1_	−0.448 ± 0.007	−0.443 ± 0.007	−0.486 ± 0.008	−0.462 ± 0.009
*c*_2_	0.028 ± 0.006	0.060 ± 0.009	0.070 ± 0.003	0.097 ± 0.013
c*_2_	0.042 ± 0.013	0.051 ± 0.014	0.074 ± 0.011	0.063 ± 0.013

c_0_, c_1_, c_2_ are the coefficients of the quadratic log-normal approximation of τ(q) (Equation 23) obtained at spatial positions x = 8, 18, 75 and 134 (in δx = 0.3 mm units), with the WTMM method of moments when using the analysing wavelet g^(3)^ (Equation 16). $c_{1}^{*}$ and $c_{2}^{*}$ are the corresponding coefficients obtained with the magnitude cumulant method (Equation 21).

#### 4.1.2. Multifractal Analysis of the Impulse Energy Data With the Method of Magnitude Cumulants

**Figure 6:**
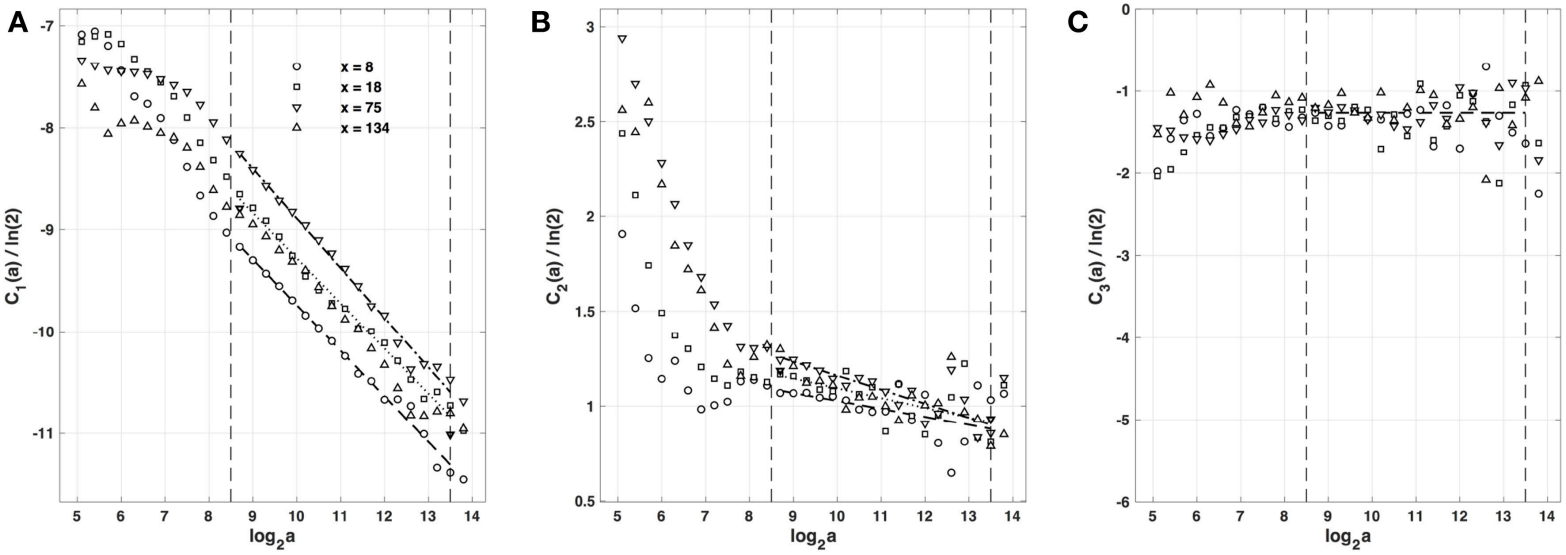
Magnitude cumulant analysis of local impulse energy time-series *E*(*x, t*) generated with our model defined in Equations (6)–(8) with the set of parameter values defined in Simul #2 (Table 1), *L* = 150 (in δ*x* = 0.3 mm units). **(A)** *C*_1_(*a*)/ln2 vs. log_2_*a*. **(B)** *C*_2_(*a*)/ln2 vs. log_2_*a*. **(C)** *C*_3_(*a*)/ln2 vs. log_2_*a*. The computation of the *C*_*n*_(*a*) (Equation 21) was performed with the third-order analyzing wavelet *g*^(3)^ (Equation 16). The vertical dashed lines delimit the range of scales (2^8.5^ ≤ *a* ≤ 2^13.5^) used for the linear regression estimate of coefficients $c_{1}^{*}$, $c_{2}^{*}$ and $c_{3}^{*}$ of τ(*q*) (Equation 22) reported in Table 1. The symbols have the same meaning as in Figures 4, 5.

As previously performed in our companion paper I (Attuel et al., 2017) for the analysis of experimental impulse energy data, we have reproduced our multifractal analysis of numerical time-series using the alternate magnitude cumulant methodology. The first-, second- and third-order cumulants where computed using Equation (21) and are plotted vs. the logarithm of the scale in Figure 6. As expected, *C*_1_(*a*), *C*_2_(*a*) and *C*_3_(*a*) display consistent scaling behavior over the same range of scales (2^8.5^ ≤ *a* ≤ 2^13.5^). The results obtained for *C*_3_(*a*) (Figure 6C) confirm that with limited 422 s long time series as recorded in experiments, there is no way to conclude about the possible departure from a log-normal quadratic τ(*q*) spectrum (*c*_3_ ≡ 0). Interestingly, the quadratic τ(*q*) spectra with parameter values $c_{1}^{*}$ and $c_{2}^{*}$ listed in Table 2, are found in good agreement with the ones previously estimated with the method of moments. This not only strengthens the multifractal diagnosis of the local impulse energy at low frequencies but it further confirms that farther from the source, larger the intermittency coefficient *c*_2_, and closer to the experimental multifractal spectra obtained in the companion paper I (Attuel et al., 2017).

### 4.2. Robustness of the Multifractal Properties of Local Impulse Energy Under Model Parameter Changes

#### 4.2.1. 1D Spatial System Length L

**Figure 7:**
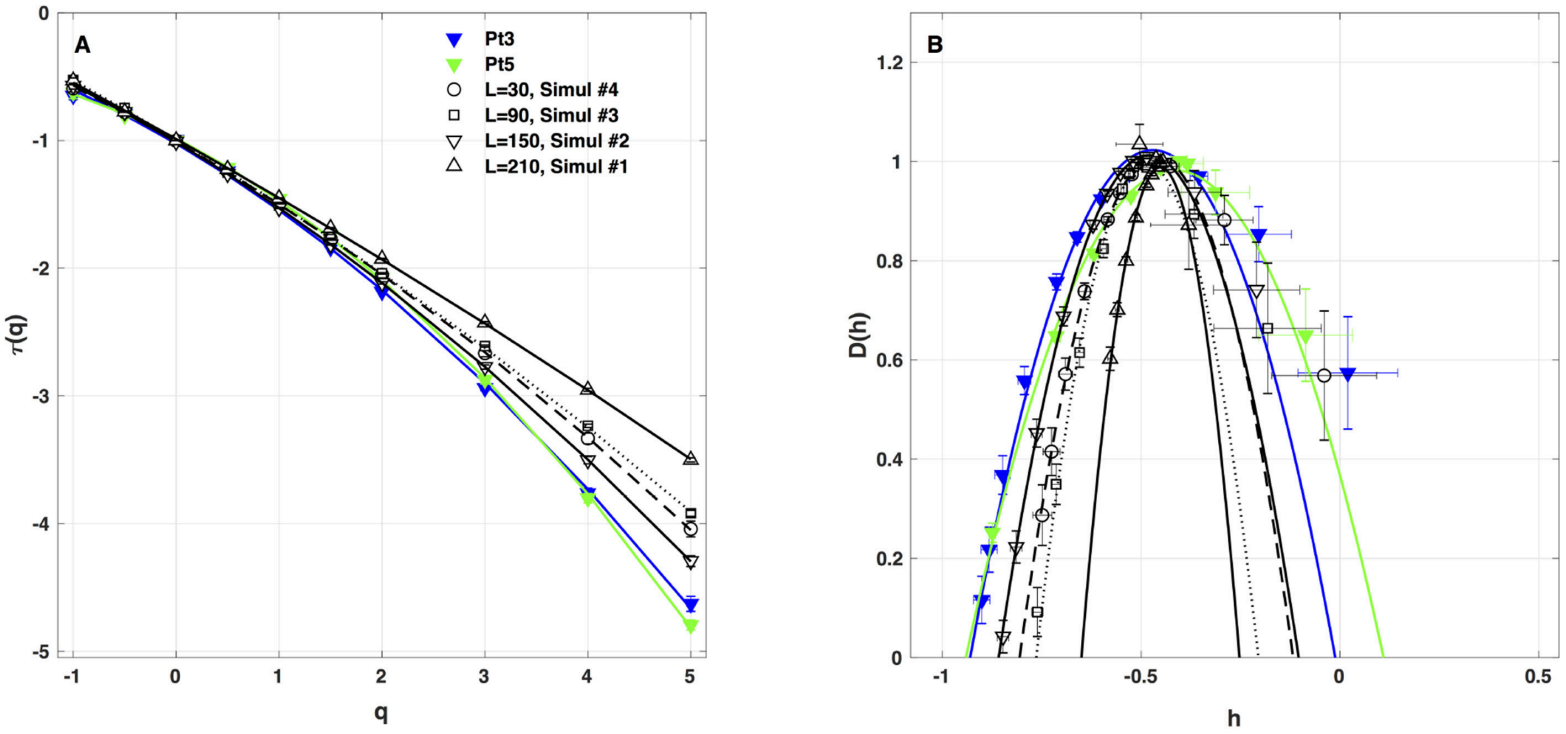
Multifractal spectra of local impulse energy time-series *E*(*x, t*) generated with our model defined in Equations (6)–(8) with the sets of parameter values defined in Table 1. **(A)** τ(*q*) vs. *q*. **(B)** *D*(*h*) vs. *h*. These spectra were computed at the same relative spatial position *x* = *L*/2 for different lengths *L* = 30 (Simul #4, ◦, ··· ), 90 (Simul #3, □, **- - - - - -** 150(Simul#2, ▽, _____), and 210 (Simul #1, △, 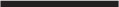) (in δ*x* = 0.3 mm units). The curves correspond to quadratic spectra (Equations 23 and 24) with parameters [*c*_0_, *c*_1_, *c*_2_] reported in Table 3. For comparison are reported the spectra previously obtained from the experimental time-series recorded at the electrodes Pt3 (blue ▼) and Pt5 (green ▼) in the left atrial posterior wall (Companion paper I Attuel et al., 2017).

As a first test of the robustness of the computed multifractal properties of local impulse energy time series, we have performed additional simulations of our 1D PDE system (Equations 6–8) for the same parameter values as before but changing the total length (in δ*x* = 0.3 mm units) of our cellular array *L* = 210 (Simul #1), 150 (Simul #2), 90 (Simul #3) and 30 (Simul #4). As shown in Figure 7 and Table 3, when using both the methods of moments and of magnitude cumulants to compute the τ(*q*) and *D*(*h*) spectra of *E*(*x* = *L*/2, *t*) at the midpoint of the array, we recover qualitatively similar multifractal spectra with *c*_0_ = *D*_*F*_ = 1, *c*_1_ ~ 0.45–0.50 and an intermittency coefficient *c*_2_ that increases when increasing *L*. This is nothing but a confirmation that *E*(*x, t*) becomes more and more intermittent when moving the spatial position *X* = *L*/2 away from the periodically beating source at *x* = 0. For 1D arrays as small as *L* = 30, *c*_2_ ~ 0.015 corresponding to a very narrow *D*(*h*) spectrum (Figure 7B). When increasing *L*, this *D*(*h*) spectrum widens progressively to become comparable to the ones obtained experimentally (*c*_2_ ~ 0.1) in Table 1 of the companion paper I (Attuel et al., 2017) for the electrodes Pt3 and Pt5 located in the left atrial posterior wall.

**Table 3:** Results of the WTMM multifractal analysis of local impulse energy time-series *E*(*x* = *L*/2, *t*) numerically simulated with our model defined in Equations (6)–(8) with the set of parameter values defined in Table 1 for different lengths: *L* = 30 (Simul #4), *L* = 90 (Simul #3), *L* = 150 (Simul #2), and *L* = 210 (Simul #1) (in δ*x* = 0.3 mm units).

	***L* = 30**	***L* = 90**	***L* = 150**	***L* = 210**
*c*_0_	1.004 ± 0.001	1.003 ± 0.000	1.013 ± 0.000	0.994 ± 0.001
*c*_1_	−0.506 ± 0.003	−0.476 ± 0.002	−0.481 ± 0.002	−0.452 ± 0.003
$c_{1}^{*}$	−0.504 ± 0.003	−0.481 ± 0.004	−0.486 ± 0.008	−0.453 ± 0.008
*c*_2_	0.012 ± 0.005	0.068 ± 0.004	0.070 ± 0.003	0.088 ± 0.005
$c_{2}^{*}$	0.024 ± 0.009	0.051 ± 0.012	0.074 ± 0.011	0.075 ± 0.009

#### 4.2.2. Fiber Conductivity κ

**Figure 8:**
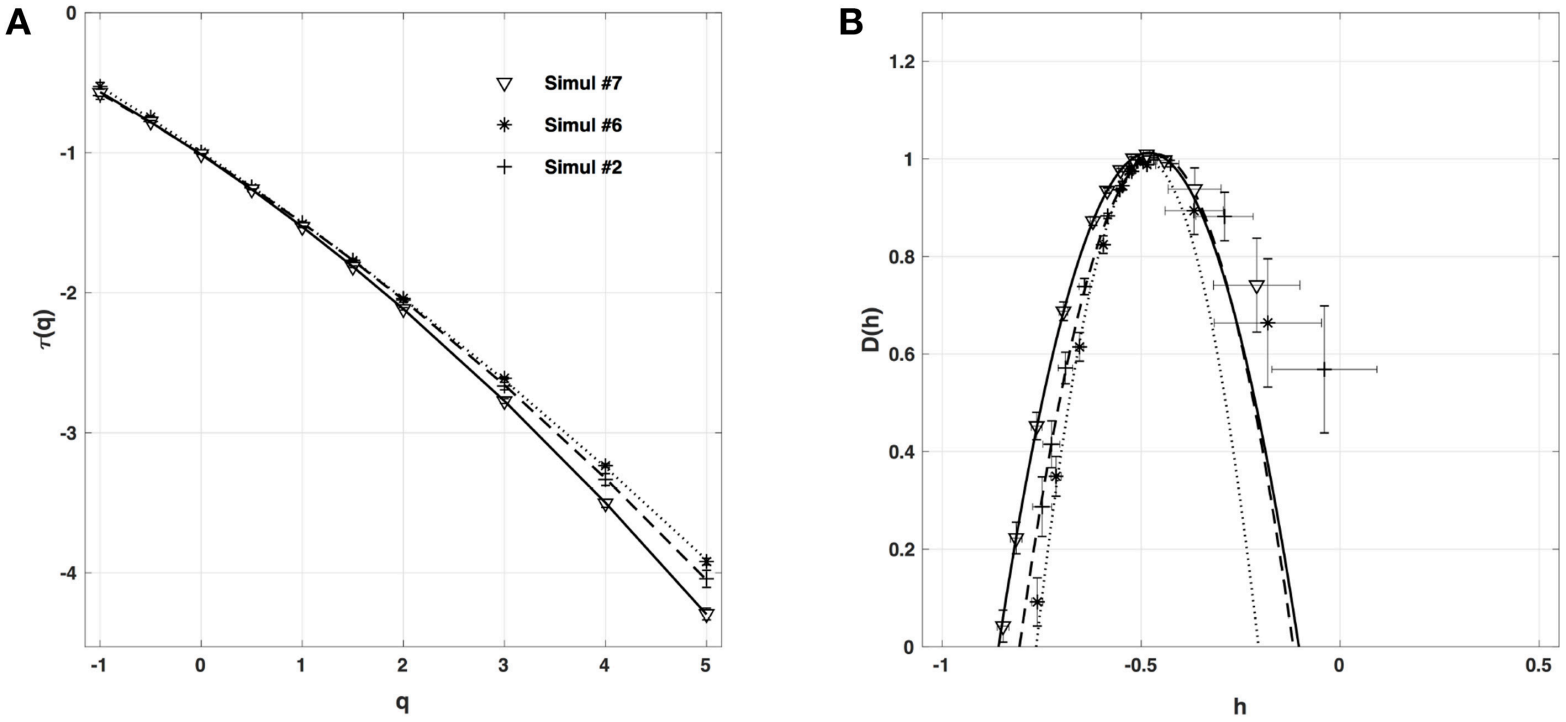
Multifractal spectra of local impulse energy time-series *E*(*x, t*) generated with our model defined in Equations (6)–(8) with the sets of parameter values defined in Simul #6 (Table 1) and *L* = 150 (in δ*x* = 0.3 mm units). **(A)** τ(*q*) vs. *q*. **(B)** *D*(*h*) vs. *h*. The spectra were computed at the same relative spatial position *x* = *L*/2 = 75 for different conductivities of the fiber: Simul #2 (▽, _____), Simul #6 (+, **- - - - -**) and Simul #7 (^*^, ··· ). The curves correspond to quadratic spectra (Equations 23 and 24) with parameters [*c*_0_, *c*_1_, *c*_2_] reported in Table 4.

When changing the fiber conductivity parameter κ that accounts for the diffusive coupling of the cells in our 1D cell array (Equation 6a), not much modification of the τ(*q*) and *D*(*h*) spectra is observed (Figure 8). Some weak narrowing of *D*(*h*) is obtained when increasing κ corresponding to a small but systematic decrease of the intermittency coefficient from *c*_2_ ~ 0.07 for κ = 0.01 mm.Ω^−1^ to *c*_2_ ~ 0.04 for κ = 0.08 mm.Ω^−1^ (Table 4). This is an indication that when strengthening the inter-cell conduction coupling, keeping all the other model parameters fixed, one somehow reduces the multifractal (intermittent) desynchronization of our 1D excitable cell network.

**Table 4:** Results of the WTMM multifractal analysis of local impulse energy time-series *E*(*x* = *L*/2, *t*) numerically simulated with our model defined in Equations (6)–(8) for different values of the conductivity parameter κ defined in Simul #2, Simul #6 and Simul #7 (Table 1), *L* = 150 (in δ*x* = 0.3 mm units).

	**κ = 0.01** mm.**Ω**^**−1**^	**κ = 0.04** mm.**Ω**^**−1**^	**κ = 0.08** mm.**Ω**^**−1**^
*c*_0_	1.013 ± 0.001	1.010 ± 0.001	0.994 ± 0.001
*c*_1_	−0.481 ± 0.003	−0.462 ± 0.004	−0.484 ± 0.005
$c_{1}^{*}$	−0.486 ± 0.008	−0.468 ± 0.010	−0.509 ± 0.009
*c*_2_	0.070 ± 0.003	0.059 ± 0.07	0.039 ± 0.009
$c_{2}^{*}$	0.074 ± 0.011	0.058 ± 0.013	0.022 ± 0.016

#### 4.2.3. New Set of Parameters

**Figure 9:**
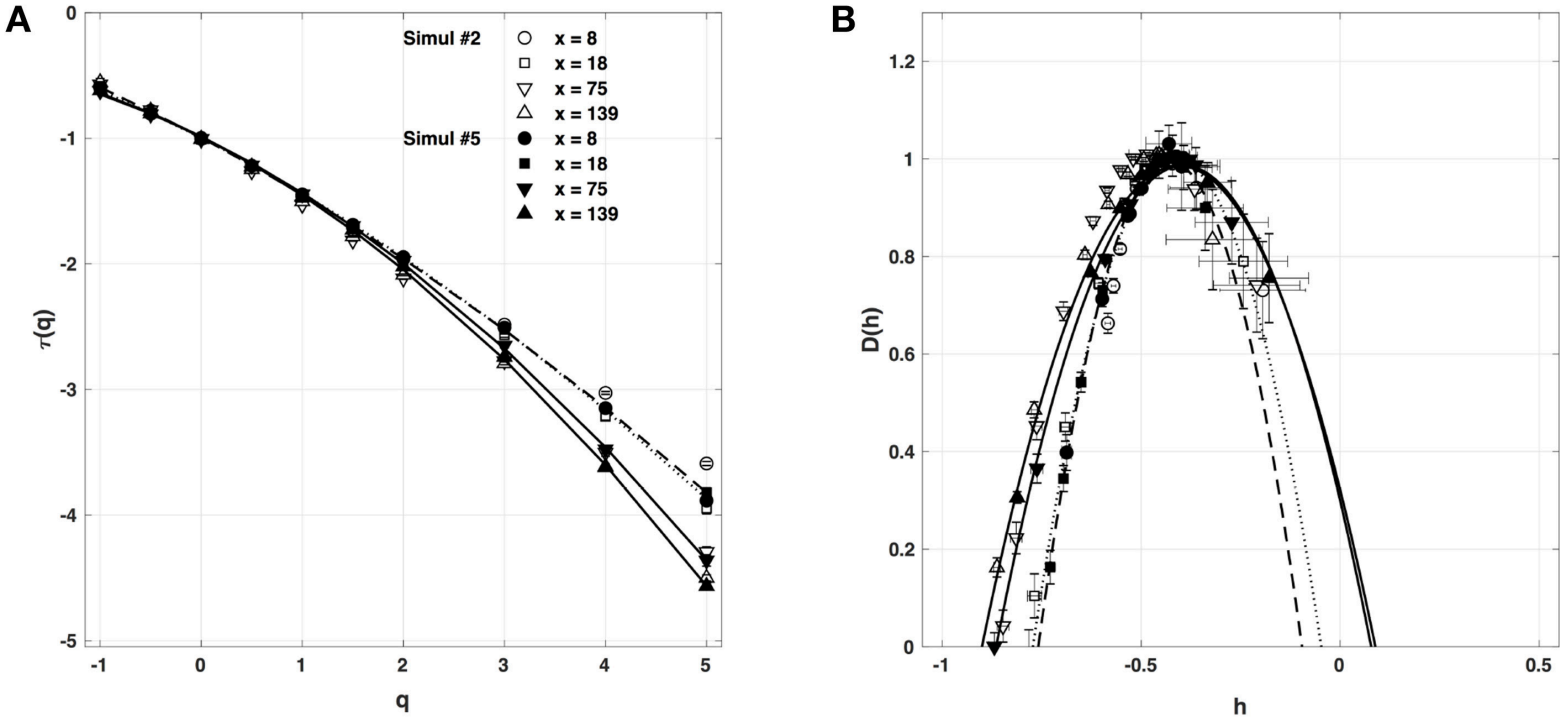
Multifractal spectra of local impulse energy time-series *E*(*x, t*) generated with our model defined in Equations (6)–(8) with the set of parameter values defined in Simul #5 (Table 1) and *L* = 150 (in δ*x* = 0.3 mm units). **(A)** τ(*q*) vs. *q*. **(B)** *D*(*h*) vs. *h*. The spectra were computed with the WTMM method with the third-order analyzing wavelet *g*^(3)^ (Equation 16). The symbols correspond to the spatial positions *x* = 8 (•, ··· ), 18 (■, **- - - - -**), 75 (▼, ___________), and 139 (▲, 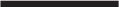) (in δ*x* = 0.3 mm units). The curves correspond to quadratic spectra (Equations 23 and 24) with parameters [*c*_0_, *c*_1_, *c*_2_] reported in Table 5. For comparison are reported in open symbols (◦, □, ▽ △), the corresponding spectra previously obtained with the set of parameter values defined in Simul #2 (Table 1) and *L* = 150 in Figure 5.

We have also reproduced the one-point multifractal analysis reported in section 4.1.1 (Simul #2) to local impulse energy numerical time-series generated with the parameter set defined in Simul #5 (Table 1) and the same 1D system spatial size *L* = 150 (in δ*x* = 0.3 mm units). Many parameters were changed, including κ, μ, ν_0_, ν_1_, γ^2^, α^2^ and ω^2^ (Table 1). As shown in Figure 9, the τ(*q*) and *D*(*h*) spectra obtained at different spatial positions *x* = 8, 18, 75, and 134 are quite similar to the ones previously obtained in Figure 5. They are robustly approximated by quadratic spectra (Equations 23 and 24, respectively) with comparable *c*_0_, *c*_1_, and *c*_2_ parameter values (Table 5).

**Table 5:** Results of the WTMM multifractal analysis of local impulse energy time-series *E*(*x, t*) numerically simulated with our model defined in Equations (6)–(8) with the parameters defined in Simul #6 (Table 1), *L* = 150 (in δ*x* = 0.3 mm units).

	***x* = 8**	***x* = 18**	***x* = 75**	***x* = 134**
*c*_0_	0.996 ± 0.001	0.994 ± 0.001	0.986 ± 0.002	0.986 ± 0.002
*c*_1_	−0.410 ± 0.006	−0.427 ± 0.002	−0.393 ± 0.008	−0.406 ± 0.009
$c_{1}^{*}$	−0.412 ± 0.006	−0.422 ± 0.007	−0.406 ± 0.003	−0.425 ± 0.005
*c*_2_	0.066 ± 0.011	0.055 ± 0.004	0.112 ± 0.016	0.124 ± 0.017
$c_{2}^{*}$	0.056 ± 0.011	0.046 ± 0.010	0.067 ± 0.010	0.070 ± 0.008

### 4.3. Two-Point Magnitude Analysis of Local Impulse Energy Data

**Figure 10:**
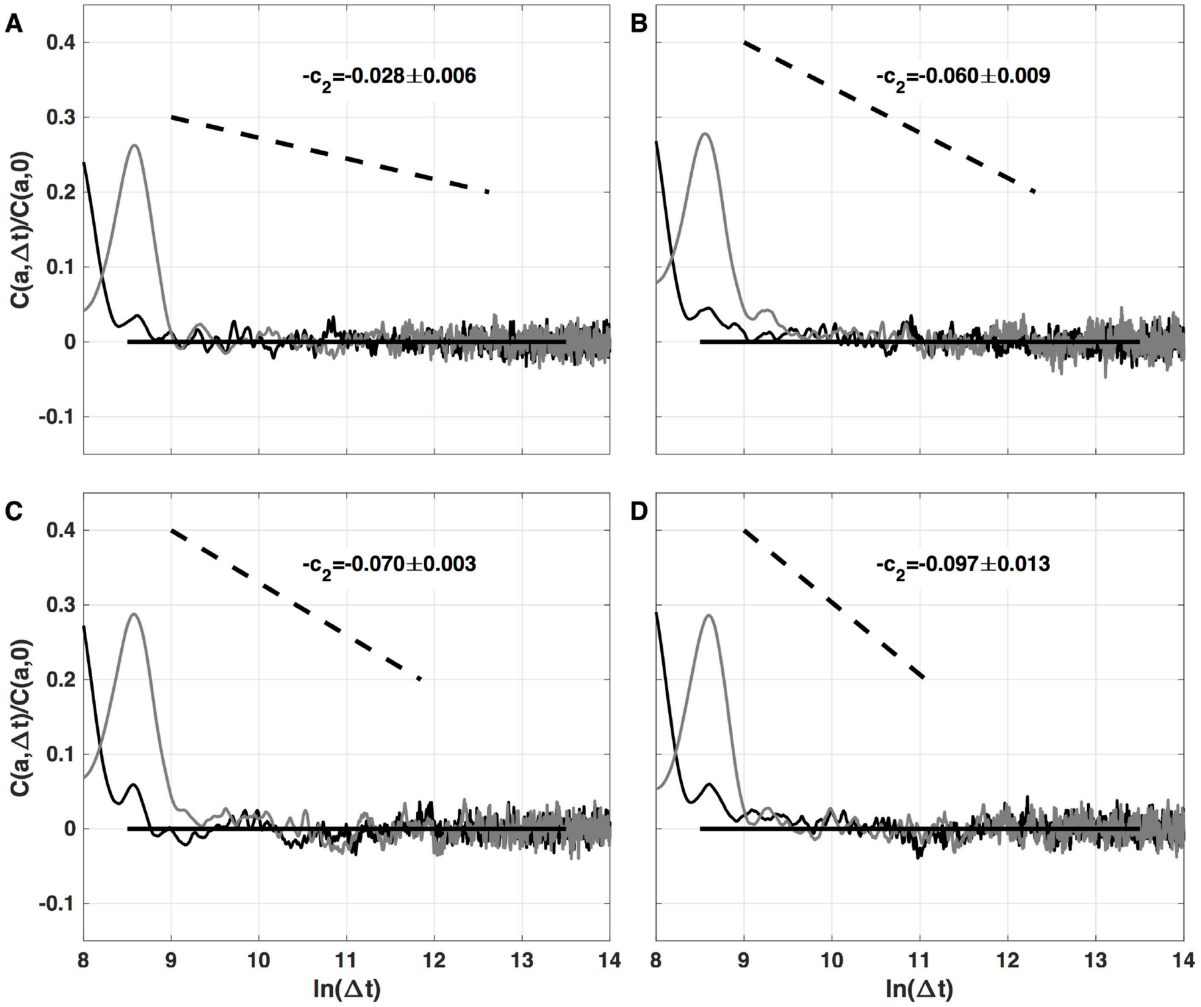
Two-point magnitude analysis of local impulse energy time-series *E*(*x, t*) generated with our model defined in Equations (6)–(8) with the sets of parameter values defined in Simul #2 (Table 1), *L* = 150 (in δ*x* = 0.3 mm units). Two-point correlation function *C*(*a*, Δ*t*)/*C*(*a*, 0) vs. ln (Δ*t*) (Equation 25) computed with the third-order analyzing wavelet *g*^(3)^ (Equation 16). The two curves correspond to scales *a* = 2^9^ (black) and 2^10^ (gray) within the scaling range. The different panels correspond to different spatial positions *x* = 8 **(A)**, 18 **(B)**, 75 **(C)**, and 134 **(D)** (in δ*x* = 0.3 mm units).

The results of the two-point magnitude correlation analysis of the local impulse energy time series numerically generated with our 1D PDE system (Equations 6–8) with the set of parameter values defined in Simul #2 are shown in Figure 10. *C*(*a*, Δ*t*)/*C*(*a*, 0) (Equation 25) computed with the third-order analyzing wavelet *g*^(3)^ (Equation 16) is represented vs. ln (Δ*t*) for two scales *a* = 2^9^ and 2^10^ in the scaling range. Strikingly, for the four numerical time-series corresponding to different spatial positions *x* = 8 (Figure 10A), 18 (Figure 10B), 75 (Figure 10C), and 134 (Figure 10D), for time-lag Δ*t* ≳ *a*, *C*(*a*, Δ)/*C*(*a*, 0) drops to zero as a clear indication that the magnitudes are uncorrelated. As a reference, we put in each panel in Figure 10, a dashed straight line of slope −*c*_2_ as predicted by Equation (26) for multifractal signals exhibiting a cascading multiplicative structure along a time-scale tree (Arneodo et al., 1998a,b). The slow decay predicted by the “multiplicative” log-normal model with intermittency coefficient *c*_2_ is definitely not observed. Thus, the numerical local impulse energy time series look much more like what has been called log-normal multifractal random noise in pioneering works to distinguish “uncorrelated” and “multiplicative” log-normal models (Arneodo et al., 1998a). Importantly, a similar absence of magnitude correlation was observed with the experimental time-series recorded at the electrodes located in the left atrial posterior wall in our companion paper I (Attuel et al., 2017). This is a strong evidence that our cardiac excitable cell network model indeed accounts for both one-point and two-point statistics of local impulse energy time-series during AF.

## 5. Conclusions

To summarize, we proposed a model of cardiac excitable cell network which accounts for the transport of AP along and across myocardial cells via the spatio-temporal interplay of voltage-gated and gap junction channels kinetics. We demonstrated that this model robustly reproduces the multifractal intermittent nature of the cardiac impulse energy experimentally recorded in the left atrial posterior wall area over times (≳ 0.5 s) longer that the mean interbeat (≃ 10^−1^ s) during AF (companion paper I, Attuel et al., 2017). In particular, this model gives full account of the experimental observation of the absence of a multiplicative time-scale structure underlying multifractal scaling. To our knowledge, our combined experimental and numerical studies are the first to report the observation, quantification and modeling of such multifractal dynamics which is found more complex than previously suspected. Preliminary exploration of the model bifurcation diagram suggests that it shares with other models a good reproducibility of the spectrum of rhythmic and AP disorders, such as early after depolarizations (EADs) or salvos of premature beats, found experimentally prior to the onset of AF. This stems here from the membrane current imbalance between depolarization and repolarization, originating in the capacitive currents building up at the GJs. In the model, the nonlinearity of the GJC temporal response was proposed to be due to a nonlinear coupling of the local electric field with the GJC charging during AP propagation. However, the nature of our studies was exploratory, with a data set limited to a few patients, and although it was performed on time-series rather long for clinical practice (422 s), they were not so long regarding the range of time scales [0.6, 10 s] where scaling was observed. The relevance of our modeling would definitely benefit of the analysis of new data over a large set of patients at different stages of AF development and to be explored in different areas of the atria. Recording electric potential time series during AF concomitantly to non intrusive Cardiovascular Magnetic Resonance (CMR) imaging could help further the assessment of atrial remodeling features such as increased expression of intercellular gap junction and conduction velocity shortening, in addition to sinus node dysfunction. Even more instructive, the comparative analysis of different types of rhythms as atrial flutter, AV junctional rhythm and various other annotated rhythms (Gadhoumi et al., 2018) would allow us to evaluate the practicality of multifractal cardiac impulse energy in the discrimination of AF from other rhythms. Also, by combining our wavelet-based multifractal analysis (low frequency) to a more classical dynamical system analysis including bifurcation diagrams, Lyapunov exponent computations, we should be in position to improve and refine our physiological heart tissue modeling and to open new perspectives toward the understanding of mechanisms of AF perpetuation.

## Author Contributions

GA, FA, HY, and AA: conception and design; GA, FA, and AA: development and methodology; GA, EG-C, FA, and AA: analysis and interpretation of data; GA, EG-C, FA, HY, and AA: writing, review, and/or revision of the manuscript; GA, EG-C, and FA: administrative, technical or material support (i.e., requiring and organizing data, constructing databases).

### Conflict of Interest Statement

The authors declare that the research was conducted in the absence of any commercial or financial relationships that could be construed as a potential conflict of interest.

## Footnotes

^1^$\sigma^{-1}=\sigma_{i}^{-1}+\sigma_{e}^{-1}$, where σ_*i*_ and σ_*e*_ are the internal and external medium conductivities in bi-domain models.
